# High monkeypox vaccine acceptance among male users of smartphone-based online gay-dating apps in Europe, 30 July to 12 August 2022

**DOI:** 10.2807/1560-7917.ES.2022.27.42.2200757

**Published:** 2022-10-20

**Authors:** Juliana Reyes-Urueña, Angelo D'Ambrosio, Roberto Croci, Benjamin Bluemel, Orlando Cenciarelli, Anastasia Pharris, Nicole Dukers-Muijrers, Will Nutland, Steph Niaupari, Jawad Badran, Gianfranco Spiteri, Teymur Noori

**Affiliations:** 1 European Centre for Disease Prevention and Control (ECDC), Stockholm, Sweden; 2 Health Promotion, CAPHRI, Maastricht University, Maastricht, the Netherlands; 3 Public Health Service South Limburg, the Netherlands; 4 The Love Tank CIC/PrEPster, London, United Kingdom; 5 Grindr/Grindr for Equality, West Hollywood, California, United States of America; 6 Hornet, Los Angeles, California, United States of America

**Keywords:** Monkeypox, Vaccination, Process, acceptance, men who have sex with men, survey

## Abstract

We assess monkeypox vaccination acceptance among male adults in the European Region. We conducted an online survey through two dating apps targeting men who have sex with men, from 30 July to 12 August 2022. We developed Bayesian hierarchical logistic regression models to investigate monkeypox vaccination acceptance. Overall crude vaccination acceptance was 82% and higher in north-western compared to south-eastern European regions. Acceptance strongly rose with perception of increased disease severity and transmission risk, and in individuals linked to healthcare.

Since March 2022, a multi-country outbreak of the monkeypox (MPX) virus has been predominantly affecting gay, bisexual and other men who have sex with men (MSM) in non-endemic countries. Data suggest that immunisation against smallpox confers a high degree of protection and immunity against MPX virus [1]. Nonetheless, vaccine hesitancy may pose an important barrier to control efforts. In this study, we assess the acceptance of MPX vaccination among male adult users of smartphone-based online gay-dating apps in the World Health Organization (WHO) European Region.

## Survey and participants

We conducted an online survey among adults ≥ 18-years old using two smartphone-based online gay-dating apps, Grindr and Hornet, in the WHO European Region. Users of both dating apps are primarily MSM. The survey was built and delivered through the European Union (EU) Survey platform [2] and translated into 25 different languages. It was advertised on the two apps, as well as through community-based organisations (CBOs) across the European Region. On the Grindr app, pop-up messages were used with a click-through rate of 6.35% on 30 July (range among 41 countries: 2.94% to 9.05%) and 5.73% on 7 August (range among 25 countries: 8.67% to 2.94%). On the Hornet app, users received an inbox message with the link of the survey, which had an overall click-through rate of 15.1% during the 30 July–12 August period. Data related to the Netherlands were collected through a web survey on other platforms according to the same protocol [3] albeit with a few questions from the app-based survey omitted (see second table’s footnote).

Among 32,902 individuals who answered the survey, the median age was 38 years (interquartile range (IQR):  30–47). Most of the respondents were living in the Western and Mediterranean subregions of Europe (Table 1 and Supplementary Material S1 where subregions of the European Region referred to in the current study are described), and 16.3% (n = 5,378) were migrants (defined as having a country of birth different from their country of residence), mostly coming from South America or another European country (Table 1 and Supplementary Material S1 and S2 for the regional categorisation, as well as S4 for sample description by region of residence).

**Table 1:** Sociodemographic characteristics of respondents of the monkeypox vaccine acceptance survey, World Health Organization European Region, 30 July–12 August 2022

Characteristic	Sample description		Vaccine acceptance^a^							
	Number	%^b^	Acceptance number	%^c^	Hesitancy number	%^c^	Refusal number	%	No answer/ missing number	%^c^
**Total**	32,902	100	26,980	82	2,890	8.8	2,686	8.2	346	1.1
**Median age in years (IQR)**	38 (30–47)		39 (30–48)		35 (27–45)		36 (28–45)		38 (31–50)	
**Age category in years**										
18–29	7,724	23.5	5,967	77.3	915	11.8	767	9.9	75	1.0
30–39	9,802	29.8	8,003	81.6	859	8.8	827	8.4	113	1.2
40–49	8,549	26.0	7,220	84.5	626	7.3	633	7.4	70	0.8
50–84	6,827	20.7	5,790	84.8	490	7.2	459	6.7	88	1.3
**Subregion of residence^d^**										
Baltics	131	0.4	106	80.9	15	11.5	10	7.6	0	0.0
Central Europe	2,628	8.0	1,760	67.0	376	14.3	472	18.0	20	0.8
Eastern Europe	1,602	4.9	1,060	66.2	274	17.1	249	15.5	19	1.2
Mediterranean Europe	11,424	34.7	9,826	86.0	856	7.5	686	6.0	56	0.5
Northern Europe	3,126	9.5	2,725	87.2	223	7.1	164	5.2	14	0.4
South-East Europe	1,901	5.8	1,284	67.5	299	15.7	302	15.9	16	0.8
Western Europe	12,090	36.7	10,219	84.5	847	7.0	803	6.6	221	1.8
**Migrants**										
No	27,524	83.7	22,400	81.4	2,532	9.2	2,329	8.5	263	1.0
Yes	5,378	16.3	4,580	85.2	358	6.7	357	6.6	83	1.5
**European subregion or other world region of origin^d^**										
Baltics	47	0.9	39	83.0	3	6.4	4	8.5	1	2.1
Caribbean	86	1.6	75	87.2	5	5.8	2	2.3	4	4.7
Central America	150	2.8	141	94.0	2	1.3	6	4.0	1	0.7
Central Europe	420	7.8	312	74.3	53	12.6	46	11.0	9	2.1
Eastern Europe	292	5.4	209	71.6	38	13.0	41	14.0	4	1.4
Eastern Mediterranean	254	4.7	212	83.5	20	7.9	17	6.7	5	2.0
Mediterranean Europe	713	13.3	617	86.5	45	6.3	44	6.2	7	1.0
Northern America	210	3.9	189	90.0	8	3.8	13	6.2	0	0.0
Northern Europe	130	2.4	115	88.5	9	6.9	6	4.6	0	0.0
South America	1,241	23.1	1,157	93.2	49	3.9	29	2.3	6	0.5
South-East Asia	67	1.2	52	77.6	9	13.4	4	6.0	2	3.0
South-East Europe	315	5.9	232	73.7	36	11.4	44	14.0	3	1.0
Sub-Saharan Africa	166	3.1	136	81.9	14	8.4	12	7.2	4	2.4
Western Europe	1,037	19.3	886	85.4	58	5.6	83	8.0	10	1.0
Western Pacific	155	2.9	142	91.6	7	4.5	3	1.9	3	1.9
Unclear region^e^	95	1.8	66	69.5	2	2.1	3	3.2	24	25.3

IQR: interquartile range.

^a ^Monkeypox vaccine acceptance and hesitancy were measured by how many respondents agreed with the following statement: ‘If the vaccine for monkeypox is offered to you, will you get vaccinated?’ A five-point Likert-type rating scale was used with the following range of choices: ‘I will get vaccinated, probably yes, not sure, probably not, I won’t get vaccinated, I don’t want to answer.’ Those who chose ‘I will get vaccinated’ or ‘Probably yes’ were defined as accepting. Those who chose ‘not sure’ were defined as hesitant. Those who chose ‘probably not’ or ‘I won’t get vaccinated’ were defined as refusal (Supplementary Material S3 for sample description for the whole five-point Likert-type rating scale).

^b^ The denominator of the percentages is the total sample for all characteristics apart from the region of origin, for which the denominator is the total number of migrant respondents.

^c^ The percentage is calculated based on the total number of participants in the same sub-category (i.e. second column of the table on the same row).

^d^ The countries considered in each European subregion are described in the Supplementary Material sections S1 and S2. Countries considered in regions outside of Europe are described in Supplementary Material S2.

^e^ The region was not clearly identifiable from the respondent answer.

Overall, 11.5% (n = 3,780) of respondents were people living with HIV (PLWHIV) on antiretroviral therapy (ART), 0.4% (n = 123) were PLWHIV not on ART (Table 2). Of those who were HIV-negative, 26.1% (7,210/27,585) among those who provided information reported using pre-exposure prophylaxis (PrEP) for HIV in the last 3 months. Almost a quarter of respondents (18.7%; n = 6,156) were diagnosed with a sexually transmitted infection (STI) in the last 12 months and 8.8% (n = 2,892) had engaged in chemsex (defined as having used mephedrone, GHB/GBL, ketamine or crystal methamphetamine during sex with other sexual partners) [4] in the last 3 months.

**Table 2:** Clinical, risk behaviour characteristics as well as perceptions, attitudes and previous monkeypox diagnoses of respondents of the monkeypox vaccine acceptance survey, World Health Organization European Region, 30 July–12 August 2022

Characteristic	Sample description		Vaccine acceptance^a^							
	Number	%^b^	Acceptance number	%^c^	Hesitancy number	%^c^	Refusal number	%^c^	No answer/ missing number	%^c^
**HIV**										
HIV −	27,585	83.8	22,538	81.7	2,467	8.9	2,303	8.3	277	1.0
HIV + on ART	3,780	11.5	3,335	88.2	209	5.5	208	5.5	28	0.7
HIV + not on ART	123	0.4	92	74.8	10	8.1	20	16.3	1	0.8
HIV status unknown	989	3.0	709	71.7	156	15.8	111	11.2	13	1.3
Prefer not to answer	339	1.0	242	71.4	40	11.8	37	10.9	20	5.9
Missing	86	0.3	64	74.4	8	9.3	7	8.1	7	8.1
**PrEP^d^**										
No	21,526	74.2	16,827	78.2	2,364	11	2,168	10.1	167	0.8
Yes	7,210	24.9	6,543	90.7	275	3.8	261	3.6	131	1.8
Missing	263	0.9	183	69.6	32	12.2	29	11.0	19	7.2
**STI diagnosis in the last 12 months**										
No	26,059	79.2	20,957	80.4	2,493	9.6	2,390	9.2	219	0.8
Yes	6,156	18.7	5,521	89.7	308	5	231	3.8	96	1.6
Unknown	474	1.4	356	75.1	65	13.7	46	9.7	7	1.5
Prefer not to answer	111	0.3	74	66.7	14	12.6	12	10.8	11	9.9
Missing	102	0.3	72	70.6	10	9.8	7	6.9	13	12.7
**Engaged in chemsex in last 3 months**										
No	29,723	90.3	24,270	81.7	2,750	9.3	2,465	8.3	238	0.8
Yes	2,892	8.8	2,503	86.5	123	4.3	195	6.7	71	2.5
Prefer not to answer	241	0.7	178	73.9	16	6.6	24	10.0	23	9.5
Missing	46	0.1	29	63.0	1	2.2	2	4.3	14	30.4
**Perception of personal risk of MPX infection**										
Not worried	4,211	12.8	2,087	49.6	630	15	1,462	34.7	32	0.8
Slightly worried	7,277	22.1	5,696	78.3	907	12.5	613	8.4	61	0.8
Moderately worried	7,232	22.0	6,221	86.0	658	9.1	295	4.1	58	0.8
Worried	8,269	25.1	7,627	92.2	390	4.7	157	1.9	95	1.1
Very worried	5,243	15.9	5,004	95.4	143	2.7	78	1.5	18	0.3
I don’t know	630	1.9	330	52.4	161	25.6	80	12.7	59	9.4
Missing	40	0.1	15	37.5	1	2.5	1	2.5	23	57.5
**Perception of MPX severity**										
Not severe	1,282	3.9	527	41.1	118	9.2	625	48.8	12	0.9
Slightly severe	3,874	11.8	2,812	72.6	443	11.4	586	15.1	33	0.9
Moderately severe	10,170	30.9	8,495	83.5	991	9.7	634	6.2	50	0.5
Severe	10,373	31.5	9,275	89.4	634	6.1	335	3.2	129	1.2
Very severe	4,586	13.9	4,275	93.2	171	3.7	106	2.3	34	0.7
I don’t know	2,581	7.8	1585	61.4	531	20.6	398	15.4	67	2.6
Missing	36	0.1	11	30.6	2	5.6	2	5.6	21	58.3
**Diagnosed with MPX (respondent or someone the respondent knows)**										
No	26,081	79.3	20,915	80.2	2,550	9.8	2,408	9.2	208	0.8
Yes, only me	232	0.7	204	87.9	7	3.0	17	7.3	4	1.7
Yes, me and someone I know	619	1.9	561	90.6	23	3.7	28	4.5	7	1.1
Yes, only someone I know	3,830	11.6	3,545	92.6	117	3.1	102	2.7	66	1.7
I don’t know	2,018	6.1	1,690	83.7	184	9.1	122	6	22	1.1
Prefer not to answer	83	0.3	53	63.9	6	7.2	7	8.4	17	20.5
Missing	39	0.1	12	30.8	3	7.7	2	5.1	22	56.4
**General perception of protection provided by vaccines against diseases^e^**										
Strongly disagree	1,293	4.0	924	71.5	92	7.1	270	20.9	7	0.5
Slightly disagree	702	2.2	262	37.3	152	21.7	280	39.9	8	1.1
Neither disagree nor agree	951	3.0	268	28.2	249	26.2	406	42.7	28	2.9
Slightly agree	7,139	22.2	5,133	71.9	1,046	14.7	919	12.9	41	0.6
Strongly agree	21,590	67.3	19,651	91	1,212	5.6	677	3.1	50	0.2
I don’t know	401	1.2	169	42.1	107	26.7	85	21.2	40	10.0
Missing	24	0.1	8	33.3	2.0	8.3	1	4.2	13	54.2
**Have you been worried about being treated differently due to monkeypox^e^**										
No	12,245	38.1	9,363	76.5	1,228	10	1,598	13.1	56	0.5
Yes	11,558	36.0	10,316	89.3	749	6.5	472	4.1	21	0.2
I don’t know	5,310	16.5	4,229	79.6	661	12.4	394	7.4	26	0.5
Prefer not to answer	257	0.8	132	51.4	38	14.8	38	14.8	49	19.1
Missing	2,730	8.5	2,375	87.0	184	6.7	136	14.8	35	1.3
**Preferred place to get vaccinated^e,f^**										
In an STI clinic	3,399	11.1	3,012	88.6	281	8.3	96	2.8	10	0.3
With my general practitioner	5,140	16.8	4,278	83.2	609	11.8	233	4.5	20	0.4
In a community-based centre	1,234	4.0	1,038	84.1	153	12.4	40	3.2	3	0.2
In a vaccination programme centre	5,493	17.9	4,645	84.6	615	11.2	215	3.9	18	0.3
It doesn’t matter	14,473	47.2	13,086	90.4	950	6.6	416	2.9	21	0.1
I don’t know	794	2.6	297	37.4	238	30	188	23.7	71	8.9
Missing	134	0.4	59	44.0	14	10.4	17	12.7	44	32.8

ART: antiretroviral therapy; HIV +: HIV-positive; HIV −: HIV-negative; MPX: monkeypox; PrEP: pre-exposure prophylaxis for HIV; STI: sexually transmitted infection.

^a^ Monkeypox vaccine acceptance and hesitancy were measured as described in the footnote ‘a’ of Table 1.

^b^ The denominator for the percentages is the total sample (n = 32,902), apart from the question related to PrEP (see footnote d) and questions not covered by the Dutch survey (see footnote e). For the PrEP question, the denominator is the number of respondents who did not declare to be HIV + (n = 28,999). For the questions not covered by the Dutch survey, the denominator is 32,100, except for the preferred place of vaccination question (n = 30,667), where the denominator also excluded those who answered ‘I won’t get vaccinated’ to the MPX vaccine acceptance question.

^c^ The percentage is calculated based on the total number of participants in the same sub-category (i.e. second column, on the same line).

^d^ This question was asked only to respondents not declaring to be HIV + (using ART or not) in the previous question.

^e^ These questions were not asked in the Dutch survey.

^f^ This question was asked only to those who did not answer ‘I won’t get vaccinated’ in the MPX vaccine acceptance question.

A total of 851 respondents (2.6%) reported that they had been diagnosed with MPX, whereas 13.5% (n = 4,449) knew someone who had been diagnosed with MPX. Overall, 45.4% (n = 14,959) thought that MPX was a ‘severe’ or ‘very severe’ disease, 56.9% (n = 18,720) were moderately, slightly, or not worried about the risk of MPX infection and 36.0% (n = 11,558) were worried about being treated differently due to MPX (Table 2).

## Monkeypox vaccination acceptance

Overall, 26,980 (82.0%) respondents reported they would accept MPX vaccination (Table 1), with 20,266 (61.6%) and 6,714 (20.4%) who would respectively surely or probably accept it (Supplementary Material S3; sample description reporting all vaccine acceptance categories). A total of 2,890 (8.8%) were hesitant and 2,686 (8.2%) would likely (1,236, 3.8%) or surely (1,450, 4.4%) refuse to get vaccinated.

We used a Bayesian nested random intercept logistic regression model [5-7] to investigate the geographical heterogeneity in MPX vaccination acceptance (Supplementary Material S5; model specification). The model adjusts the estimates taking into consideration the aggregation of the respondents into countries and European subregions (Supplementary Material S1), achieving more robust estimates, including for countries with fewer respondents, by pooling information from geographical neighbours [8,9]. We reported the median posterior acceptance and the 90% credible intervals (90% CrI) [10,11].

**Figure 1:**
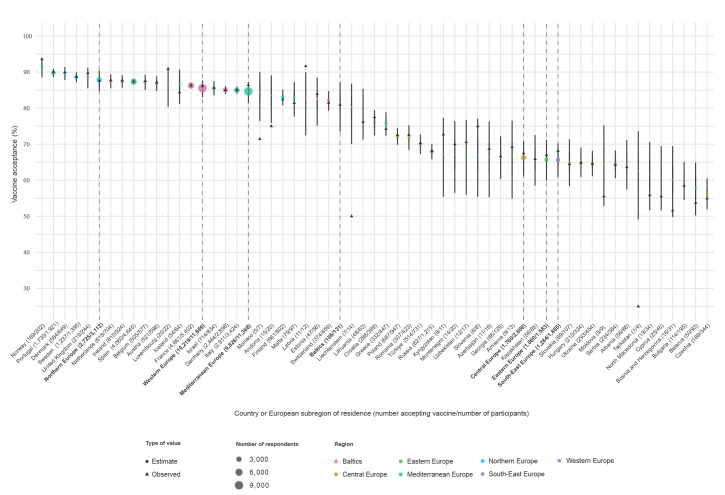
Monkeypox vaccination acceptance by subregion and country of residence, World Health Organization European Region, 30 July–12 August 2022

The general estimated acceptance of MPX vaccination in our survey, after adjusting for the participants’ country of residence had a 90% CrI ranging between 68% and 85%. We observed a clear geographical gradient (Figure 1 and Supplementary Material S6; baseline MPX vaccine acceptance probabilities), with higher acceptance in Northern (90% CrI: 84.8%–90.4%), and Western Europe (90% CrI: 83.1%–87.7%) and lower acceptance in South-East (90% CrI: 60.9%–70.2%) and Eastern Europe (90% CrI: 59.9%–71.1%).

Modelled estimates (with 90% CrI) and observed rates of vaccine acceptance are presented for each participating country, coloured by subregion. Estimates at the subregional level are also reported and highlighted by vertical dashed lines for easier identification. Point sizes are proportional to the number of respondents in each country/subregion. The modelled and the observed rates differences are due to the effect of using a hierarchical random intercept model, which reduces the contribution of observed rates if derived from countries with a small number of respondents, shifting them toward the subregional mean.

## Factors associated with vaccination

**Figure 2:**
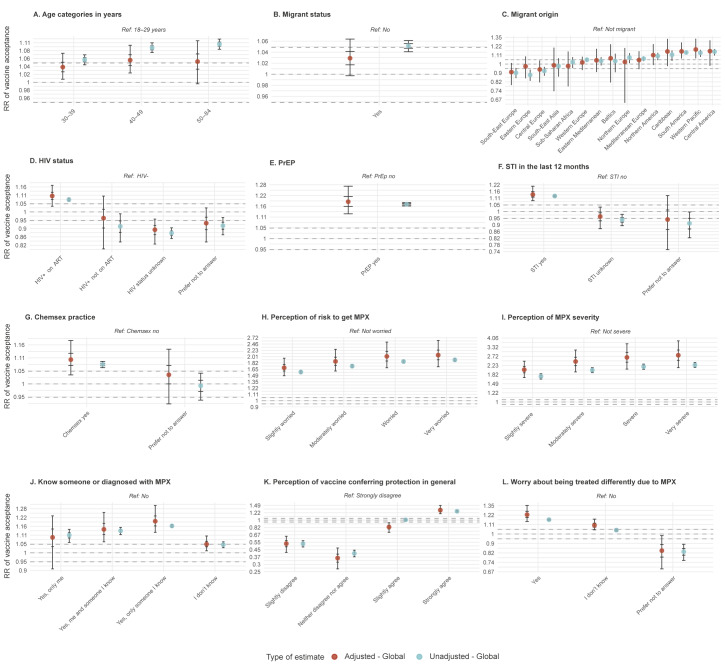
Determinants of monkeypox vaccination acceptance stratified by subregion in the World Health Organization European Region, 30 July–12 August 2022

ART: antiretroviral therapy; CrI: credible interval; MPX: monkeypox; PrEP: pre-exposure prophylaxis for HIV; ref.: reference category; RR: relative risk; STI: sexually transmitted infection.

A similar model was used to investigate the association between factors collected in the survey and vaccination acceptance, with and without adjusting for country and European subregion of residence through random effects. We reported the median posterior unadjusted (RR) and adjusted (aRR) vaccine acceptance relative risks and their 90% CrI (Figure 2 and Supplemental Material S7; factor association with MPX vaccine acceptance).

For each answer, the estimated RR (with 50% and 90% CrI) are reported related to the change in MPX vaccine acceptance compared with a reference group (reported in the plot titles). Estimates are generated by models both adjusted (red points) and unadjusted (turquoise points) for the geographical heterogeneity (i.e. using or not the nested subregion and country random effects). More details about factor association by country and subregion can be found in the Supplementary Material S8, S9, S10.

Vaccination acceptance was strongly associated with the belief that MPX is a ‘severe’ (aRR: 2.65; 90% CrI: 2.05–3.58) or ‘very severe’ (aRR: 2.78; 90% CrI: 2.12–3.81) disease, vs ‘not severe’ respectively. Moreover, acceptance was also strongly related to being worried (aRR:  2.02; 90% CrI: 1.69–2.54) and very worried (aRR:  2.06; 90% CrI: 1.72–2.61) about the risk of acquiring MPX infection, vs ‘not worried’ respectively (Figure 2). Having encountered the disease was associated with more acceptance, especially when encountered within a person’s own social network, such as ‘me and someone I know’ (aRR:  1.14; 90% CrI: 1.06–1.26) and ‘only someone I know’ (aRR:  1.2; 90% CrI: 1.12–1.31), vs ‘know no one with MPX’ respectively (Figure 2).

Vaccination acceptance was higher among older respondents (84.5% for 40 to 49 year-olds and 84.8% for 50 to 84 year-olds vs 77.3% for 18 to 30 year-olds, see Table 1) but the difference became much less relevant once adjusting for the respondent residence (Figure 2), reflecting the confounding due to North-Western European countries having both higher acceptance and older mean age. Nevertheless, some potential effect of age could still be seen for respondents in South-East and Central Europe, where older MSMs showed a moderately higher acceptance (Supplementary Material S9). Being migrant did not show a relevant overall association (Figure 2); however, when stratified by regions of origin, being migrant seemed to be associated with higher (Americas and Western Pacific regions) or lower (South-East Europe subregion) acceptance compared with non-migrants (Figure 2, Supplementary Material S9).

Being linked to healthcare was positively associated with vaccine acceptance:  PLWHIV on ART reported a slightly higher vaccine acceptance compared with HIV-negative individuals (88.2% vs 81.7%; aRR:  1.1; 90% CrI: 1.03–1.17); the association with vaccine acceptance was stronger among HIV-negative people on PrEP vs those not on PrEP (90.7% vs 78.2%; aRR:  1.19; 90% CrI: 1.12–1.28).

Those considered to have higher risk sexual behaviours had higher vaccine acceptance rates, such as having been diagnosed with an STI in the last 12 months (89.7% vs 80.4%; aRR:  1.14; 90% CrI: 1.08–1.21) and engaging in chemsex in the last 3 months (86.5% vs 81.7%; aRR:  1.1; 90% CrI: 1.03–1.18). Conversely, being PLWHIV not on ART had lower vaccine acceptance (74.8% vs 88.2% PLWHIV on ART), however, the number of responses was too low (n = 123) to draw definitive conclusions (aRR: 0.96; 90% CrI: 0.8–1.1) especially once adjusting for region of residency.

Respondents who reported worry about being treated differently due to MPX were more likely to accept being vaccinated (89.3% vs 76.5%; aRR: 1.23; 90% CrI: 1.14–1.35).

## Discussion

In this sample of MSM using smartphone-based online gay-dating apps in the WHO European Region the acceptance of MPX vaccination was high. After adjustment by country and region of residence, we found that those with high perception of susceptibility to MPX infection, beliefs that MPX is a severe disease, and worries about being treated differently due to MPX were more willing to accept MPX vaccination. These are key associated factors in determining the decision to be vaccinated, as have been described in other studies [12,13].

Our results also suggest important geographical differences in MPX vaccination acceptance, with lower rates reported by people living in South-Eastern and Central Europe as well as the Eastern European subregion. MPX vaccination acceptance might be influenced by the region of origin, as, at least from what we observe for migrants of European origin, people born outside their country of residence tend to have a similar level of acceptance to their region of birth, despite living in a region with a different level of vaccine acceptance.

Being linked to routine healthcare, for example PLWHIV on ART, HIV-negative persons on PrEP or having a recent STI diagnosis, was a positive predictor of willingness to get vaccinated. Such an association with an existing healthcare engagement was also found in a United States study, which showed a higher vaccination rate among MSM on HIV PrEP or who were recently tested for STIs [14]. Increasing HIV prevalence among MPX cases over time suggests that MPX might be increasingly transmitted among networks of persons with HIV infection [15]. The prioritisation of people linked with healthcare might be beneficial, especially with the current shortage of vaccines, as they are easier to reach and might be an important group in transmission dynamics.

Implementing an equitable MPX vaccination strategy means meeting the needs of those groups with the highest vaccine hesitancy, such as young MSM, PLWHIV who are not on ART or those with lower perception of risk of infection and for whom the links with community-based organisations and the healthcare system are weaker. Focusing on such groups is especially relevant in regions with low general acceptance levels. The health-seeking behaviours of these vulnerable groups may differ considerably compared with the other MSM subgroups, and thus health promotion efforts, as well as services, need to be tailored to meet their needs.

CBOs in Europe have experience in delivering HIV, STI and viral hepatitis health services to the MSM community [16]. Public health providers should partner with CBOs in efforts to engage with the community to educate and promote uptake of MPX vaccination. These partnerships should also be used to facilitate access to MPX vaccination at sex-on-premises venues that cater to MSM at higher risk for MPX (those engaging in chemsex and those having been diagnosed with an STI in the last 12 months) [17]. Risk communication and community engagement (RCCE) activities need to be intensified in regions with low vaccine acceptance, as part of the core public health intervention contributing to emergency response, tailored with the implementation of a MPX vaccine strategy that addresses sexual health literacy, vaccination access and structural inequities.

The findings in this report are subject to several limitations. First, this survey represents a convenience sample of MSM using smartphone-based online dating apps who chose to participate in a survey about MPX. MSM using dating apps may have higher risk behaviours compared with those not using dating apps [18]. People at self-perceived higher risk for the disease may be more prone to getting vaccinated against it which could lead to overestimates in vaccine acceptance. Second, survey completion via a dating app necessitates a degree of digital literacy among the respondents and consequently our results may not capture views of people at risk of MPX with low levels of digital literacy. Third, these data are self-reported and might be subject to response bias (e.g. social desirability bias). Fourth, it is important to take into consideration that we did not collect socioeconomic data and are therefore not able to infer their impact on MPX vaccine acceptance. Fifth, stigma was not a focus of the survey to limit the length of the survey.

## Conclusions

Acceptance of MPX vaccination is high among MSM who use dating apps. Attention is needed to increase vaccination acceptance in some subregions of Europe and groups with indications of lower acceptance. MPX vaccination strategies which are part of a combined prevention approach for strengthening prevention services and increasing equitable vaccination access, engaging community, healthcare providers, civil society and public health professionals will promote MPX control in the WHO European Region.

## Supplementary Data

247000 Supplementary Material
