# Supplementary material for: High monkeypox vaccine acceptance among male users of smartphone-based online gay-dating apps in Europe, 30 July to 12 August 2022
Source: Euro Surveill. 2022 Oct 20;27(42):2200757. doi: 10.2807/1560-7917.ES.2022.27.42.2200757 (PMC9585877; doi:10.2807/1560-7917.ES.2022.27.42.2200757)
Supplement: Supplementary Material [file 2200757_SupplementaryMaterial.pdf]

Supplemental Material

Table of contents

S1. European subregion of residence subdivision 2
S2. European subregion and world region of origin subdivision 3
S3. Sample description reporting all vaccine acceptance categories 5
S4. Description of the sample by region 9
Central Europe 9
South-East Europe 11
Baltics 13
Eastern Europe 15
Mediterranean Europe 17
Northern Europe 19
Western Europe 22
S5. Model specification 25
S6. Baseline MPX vaccine acceptance probabilities. 28
S7. Factor association with MPX vaccine acceptance. 30
S8. Factor association with MPX vaccine acceptance by European subregion and country. 34
S9. Association plot 51
General plot 51
Migrant birth region plot 52
S10. Session Info 53
References 54

This supplementary material is hosted by Eurosurveillance as supporting information alongside the article "High monkeypox vaccine acceptance among male users of smartphone-based online gay-dating apps in Europe, 30 July to 12 August 2022" on behalf of the authors who remain responsible for the accuracy and appropriateness of the content. The same standards for ethics, copyright, attributions and permissions as for the article apply. Supplements are not edited by Eurosurveillance and the journal is not responsible for the maintenance of any links or email addresses provided therein.

S1. European subregion of residence subdivision

Respondent's countries of residence were grouped into European subregions to account for cultural similarities and compensate for noisy estimates for countries with a low number of responses to the survey.

Central Europe:

Czechia, Hungary, Poland, Republic of Moldova, Romania, Slovakia, Ukraine.

South-East Europe:

Albania, Bosnia and Herzegovina, Bulgaria, Croatia, Cyprus, Montenegro, North Macedonia, Serbia, Slovenia, Türkiye.

Baltics:

Estonia, Latvia, Lithuania.

Eastern Europe:

Armenia, Azerbaijan, Belarus, Georgia, Kazakhstan, Kyrgyzstan, Russia, Tajikistan, Uzbekistan.

Mediterranean Europe:

Greece, Israel, Italy, Malta, Portugal, Spain.

Northern Europe:

Denmark, Finland, Iceland, Norway, Sweden.

Western Europe:

Andorra, Austria, Belgium, France, Germany, Ireland, Liechtenstein, Luxembourg, Monaco, Netherlands, Switzerland, United Kingdom.

S2. European subregion and world region of origin subdivision

A similar approach was used for the countries of origin/birth.

Baltics:

Estonia, Latvia, Lithuania.

Caribbean:

Antigua and Barbuda, Aruba, Bahamas, Barbados, Cuba, Dominica, Dominican Republic, Haiti, Jamaica, Trinidad and Tobago.

Central America:

Belize, Costa Rica, El Salvador, Guatemala, Honduras, Mexico, Nicaragua, Panama.

Central Europe:

Czechia, Hungary, Poland, Republic of Moldova, Romania, Slovakia, Ukraine.

Eastern Europe:

Armenia, Azerbaijan, Belarus, Georgia, Kazakhstan, Kyrgyzstan, Russia, Tajikistan, Turkmenistan, Uzbekistan.

Eastern Mediterranean:

Afghanistan, Algeria, Bahrain, Djibouti, Egypt, Iran, Iraq, Jordan, Kuwait, Lebanon, Libya, Morocco, Pakistan, Saudi Arabia, Syrian Arab Republic, Tunisia, United Arab Emirates, Yemen.

Mediterranean Europe:

Greece, Israel, Italy, Malta, Portugal, San Marino, Spain.

Northern America:

Canada, United States of America.

Northern Europe:

Denmark, Finland, Iceland, Norway, Sweden.

South America:

Argentina, Bolivia, Brazil, Chile, Colombia, Ecuador, Paraguay, Peru, Suriname, Uruguay, Venezuela.

South-East Asia:

Bangladesh, India, Indonesia, Maldives, North Korea, Sri Lanka, Thailand, Timor-Leste.

South-East Europe:

Albania, Bosnia and Herzegovina, Bulgaria, Croatia, Cyprus, Montenegro, North Macedonia, Serbia, Slovenia, Türkiye.

Sub-Saharan Africa:

Angola, Benin, Burkina Faso, Burundi, Cabo Verde, Cameroon, Central African Republic, Comoros, Congo, Côte D'Ivoire, Democratic Republic of the Congo, Equatorial Guinea, Eritrea, Eswatini, Ethiopia, Gabon, Ghana, Guinea, Guinea Bissau, Kenya, Liberia, Madagascar, Mali, Mauritius, Mozambique, Nigeria, Rwanda, Sao Tome and Principe, Senegal, Somalia, South Africa, South Sudan, Togo, Uganda, Zambia, Zimbabwe.

Western Europe:

Andorra, Austria, Belgium, France, Germany, Ireland, Liechtenstein, Luxembourg, Monaco, Netherlands, Switzerland, United Kingdom.

Western Pacific:

Australia, Brunei Darussalam, China, Fiji, Japan, Laos, Malaysia, Mongolia, New Zealand, Papua New Guinea, Philippines, Singapore, South Korea, Viet Nam.

S3. Sample description reporting all vaccine acceptance categories

We describe the sample reporting all items the vaccine acceptance question (i.e., the five-point Likert-type rating scale plus unwillingnes to answer and missing answers). In the manuscript text we collapsed the responses in four group for easier readability. See the manuscript for the description of the table.

Description of the sample using all vaccine acceptance question items.

		MPX Vaccine acceptance						
**Question**	**Sample Description**	**I will get vaccinated**	**Probably yes**	**Not sure**	**Probably not**	**I won't get vaccinated**	**Prefer not to answer**	**Missing**
**Total**, N (%)	32902 (100)	20266 (61.6)	6714 (20.4)	2890 (8.8)	1236 (3.8)	1450 (4.4)	145 (0.4)	201 (0.6)
**Preferred place to get vaccinated**, N (%)								
In an STI clinic	3399 (11.1)	2344 (69)	668 (19.7)	281 (8.3)	96 (2.8)	0 (0)	10 (0.3)	0 (0)
With my general practitioner	5140 (16.8)	2969 (57.8)	1309 (25.5)	609 (11.8)	233 (4.5)	0 (0)	20 (0.4)	0 (0)
In a community-based centre	1234 (4)	693 (56.2)	345 (28)	153 (12.4)	40 (3.2)	0 (0)	3 (0.2)	0 (0)
In a vaccination program centre	5493 (17.9)	3123 (56.9)	1522 (27.7)	615 (11.2)	215 (3.9)	0 (0)	16 (0.3)	2 (0)
It doesn't matter	14473 (47.2)	10487 (72.5)	2599 (18)	950 (6.6)	416 (2.9)	0 (0)	21 (0.1)	0 (0)
I don't know	794 (2.6)	137 (17.3)	160 (20.2)	238 (30)	188 (23.7)	0 (0)	71 (8.9)	0 (0)
Missing	134 (0.4)	44 (32.8)	15 (11.2)	14 (10.4)	17 (12.7)	0 (0)	4 (3)	40 (29.9)
**Age**, Median (IQR)	38.0 (30 - 47)	39.0 (31 - 48)	37.0 (28 - 47)	35.0 (27 - 45)	36.0 (28 - 45)	36.5 (28 - 45)	35.0 (28 - 44)	40.0 (31 - 51)
**Age category**, N (%)								
18-29	7724 (23.5)	4083 (52.9)	1884 (24.4)	915 (11.8)	366 (4.7)	401 (5.2)	42 (0.5)	33 (0.4)
30-39	9802 (29.8)	6100 (62.2)	1903 (19.4)	859 (8.8)	389 (4)	438 (4.5)	46 (0.5)	67 (0.7)
40-49	8549 (26)	5618 (65.7)	1602 (18.7)	626 (7.3)	258 (3)	375 (4.4)	32 (0.4)	38 (0.4)
50-84	6827 (20.7)	4465 (65.4)	1325 (19.4)	490 (7.2)	223 (3.3)	236 (3.5)	25 (0.4)	63 (0.9)
**Region of residence**, N (%)								
Baltics	131 (0.4)	74 (56.5)	32 (24.4)	15 (11.5)	6 (4.6)	4 (3.1)	0 (0)	0 (0)
Central Europe	2628 (8)	1048 (39.9)	712 (27.1)	376 (14.3)	216 (8.2)	256 (9.7)	16 (0.6)	4 (0.2)
Eastern Europe	1602 (4.9)	529 (33)	531 (33.1)	274 (17.1)	87 (5.4)	162 (10.1)	17 (1.1)	2 (0.1)
Mediterranean Europe	11424 (34.7)	7723 (67.6)	2103 (18.4)	856 (7.5)	293 (2.6)	393 (3.4)	47 (0.4)	9 (0.1)
Northern Europe	3126 (9.5)	1981 (63.4)	744 (23.8)	223 (7.1)	99 (3.2)	65 (2.1)	8 (0.3)	6 (0.2)
South-East Europe	1901 (5.8)	838 (44.1)	446 (23.5)	299 (15.7)	124 (6.5)	178 (9.4)	13 (0.7)	3 (0.2)
Western Europe	12090 (36.7)	8073 (66.8)	2146 (17.8)	847 (7)	411 (3.4)	392 (3.2)	44 (0.4)	177 (1.5)
**Migrants**, N (%)								

Description of the sample using all vaccine acceptance question items. *(continued)*

Question	Sample Description	I will get vaccinated	Probably yes	Not sure	Probably not	I won't get vaccinated	Prefer not to answer	Missing
No	27524 (83.7)	16567 (60.2)	5833 (21.2)	2532 (9.2)	1069 (3.9)	1260 (4.6)	111 (0.4)	152 (0.6)
Yes	5378 (16.3)	3699 (68.8)	881 (16.4)	358 (6.7)	167 (3.1)	190 (3.5)	34 (0.6)	49 (0.9)
**Migrant Origin**, N (%)								
Baltics	47 (0.9)	31 (66)	8 (17)	3 (6.4)	3 (6.4)	1 (2.1)	1 (2.1)	0 (0)
Caribbean	86 (1.6)	64 (74.4)	11 (12.8)	5 (5.8)	1 (1.2)	1 (1.2)	2 (2.3)	2 (2.3)
Central America	150 (2.8)	129 (86)	12 (8)	2 (1.3)	3 (2)	3 (2)	0 (0)	1 (0.7)
Central Europe	420 (7.8)	232 (55.2)	80 (19)	53 (12.6)	19 (4.5)	27 (6.4)	8 (1.9)	1 (0.2)
Eastern Europe	292 (5.4)	126 (43.2)	83 (28.4)	38 (13)	21 (7.2)	20 (6.8)	4 (1.4)	0 (0)
Eastern Mediterranean	254 (4.7)	172 (67.7)	40 (15.7)	20 (7.9)	7 (2.8)	10 (3.9)	4 (1.6)	1 (0.4)
Mediterranean Europe	713 (13.3)	506 (71)	111 (15.6)	45 (6.3)	19 (2.7)	25 (3.5)	1 (0.1)	6 (0.8)
Northern America	210 (3.9)	158 (75.2)	31 (14.8)	8 (3.8)	5 (2.4)	8 (3.8)	0 (0)	0 (0)
Northern Europe	130 (2.4)	89 (68.5)	26 (20)	9 (6.9)	2 (1.5)	4 (3.1)	0 (0)	0 (0)
South America	1241 (23.1)	988 (79.6)	169 (13.6)	49 (3.9)	13 (1)	16 (1.3)	4 (0.3)	2 (0.2)
South-East Asia	67 (1.2)	37 (55.2)	15 (22.4)	9 (13.4)	1 (1.5)	3 (4.5)	2 (3)	0 (0)
South-East Europe	315 (5.9)	169 (53.7)	63 (20)	36 (11.4)	18 (5.7)	26 (8.3)	2 (0.6)	1 (0.3)
Sub-Saharan Africa	166 (3.1)	110 (66.3)	26 (15.7)	14 (8.4)	5 (3)	7 (4.2)	3 (1.8)	1 (0.6)
Western Europe	1037 (19.3)	723 (69.7)	163 (15.7)	58 (5.6)	45 (4.3)	38 (3.7)	1 (0.1)	9 (0.9)
Western Pacific	155 (2.9)	112 (72.3)	30 (19.4)	7 (4.5)	2 (1.3)	1 (0.6)	2 (1.3)	1 (0.6)
Unclear Region	95 (1.8)	53 (55.8)	13 (13.7)	2 (2.1)	3 (3.2)	0 (0)	0 (0)	24 (25.3)
**HIV**, N (%)								
HIV-	27585 (83.8)	16732 (60.7)	5806 (21)	2467 (8.9)	1095 (4)	1208 (4.4)	105 (0.4)	172 (0.6)
HIV+ on ART	3780 (11.5)	2764 (73.1)	571 (15.1)	209 (5.5)	79 (2.1)	129 (3.4)	9 (0.2)	19 (0.5)
HIV+ not on ART	123 (0.4)	71 (57.7)	21 (17.1)	10 (8.1)	5 (4.1)	15 (12.2)	1 (0.8)	0 (0)
HIV status unknown	989 (3)	469 (47.4)	240 (24.3)	156 (15.8)	46 (4.7)	65 (6.6)	11 (1.1)	2 (0.2)
Prefer not to answer	339 (1)	177 (52.2)	65 (19.2)	40 (11.8)	8 (2.4)	29 (8.6)	19 (5.6)	1 (0.3)
Missing	86 (0.3)	53 (61.6)	11 (12.8)	8 (9.3)	3 (3.5)	4 (4.7)	0 (0)	7 (8.1)
**PrEP**, N (%)								
PrEp no	21526 (74.2)	11554 (53.7)	5273 (24.5)	2364 (11)	1030 (4.8)	1138 (5.3)	104 (0.5)	63 (0.3)
PrEP yes	7210 (24.9)	5742 (79.6)	801 (11.1)	275 (3.8)	111 (1.5)	150 (2.1)	25 (0.3)	106 (1.5)
Missing	263 (0.9)	135 (51.3)	48 (18.3)	32 (12.2)	11 (4.2)	18 (6.8)	6 (2.3)	13 (4.9)
**STI in the last 12 months**, N (%)								
STI no	26059 (79.2)	15263 (58.6)	5694 (21.9)	2493 (9.6)	1099 (4.2)	1291 (5)	110 (0.4)	109 (0.4)
STI yes	6156 (18.7)	4669 (75.8)	852 (13.8)	308 (5)	108 (1.8)	123 (2)	17 (0.3)	79 (1.3)
STI unknown	474 (1.4)	227 (47.9)	129 (27.2)	65 (13.7)	26 (5.5)	20 (4.2)	7 (1.5)	0 (0)
Prefer not to answer	111 (0.3)	55 (49.5)	19 (17.1)	14 (12.6)	1 (0.9)	11 (9.9)	10 (9)	1 (0.9)

Description of the sample using all vaccine acceptance question items. *(continued)*

Question	Sample Description	I will get vaccinated	Probably yes	Not sure	Probably not	I won't get vaccinated	Prefer not to answer	Missing
Missing	102 (0.3)	52 (51)	20 (19.6)	10 (9.8)	2 (2)	5 (4.9)	1 (1)	12 (11.8)
**Chemsex users**, N (%)								
Chemsex no	29723 (90.3)	17988 (60.5)	6282 (21.1)	2750 (9.3)	1165 (3.9)	1300 (4.4)	117 (0.4)	121 (0.4)
Chemsex yes	2892 (8.8)	2117 (73.2)	386 (13.3)	123 (4.3)	66 (2.3)	129 (4.5)	8 (0.3)	63 (2.2)
Prefer not to answer	241 (0.7)	136 (56.4)	42 (17.4)	16 (6.6)	4 (1.7)	20 (8.3)	20 (8.3)	3 (1.2)
Missing	46 (0.1)	25 (54.3)	4 (8.7)	1 (2.2)	1 (2.2)	1 (2.2)	0 (0)	14 (30.4)
**Perception of risk to get MPX**, N (%)								
Not worried	4211 (12.8)	1212 (28.8)	875 (20.8)	630 (15)	523 (12.4)	939 (22.3)	20 (0.5)	12 (0.3)
Slightly worried	7277 (22.1)	3657 (50.3)	2039 (28)	907 (12.5)	373 (5.1)	240 (3.3)	21 (0.3)	40 (0.5)
Moderately worried	7232 (22)	4520 (62.5)	1701 (23.5)	658 (9.1)	183 (2.5)	112 (1.5)	16 (0.2)	42 (0.6)
Worried	8269 (25.1)	6173 (74.7)	1454 (17.6)	390 (4.7)	88 (1.1)	69 (0.8)	19 (0.2)	76 (0.9)
Very worried	5243 (15.9)	4498 (85.8)	506 (9.7)	143 (2.7)	37 (0.7)	41 (0.8)	12 (0.2)	6 (0.1)
I don't know	630 (1.9)	194 (30.8)	136 (21.6)	161 (25.6)	31 (4.9)	49 (7.8)	57 (9)	2 (0.3)
Missing	40 (0.1)	12 (30)	3 (7.5)	1 (2.5)	1 (2.5)	0 (0)	0 (0)	23 (57.5)
**Perception of MPX severity**, N (%)								
Not severe	1282 (3.9)	392 (30.6)	135 (10.5)	118 (9.2)	127 (9.9)	498 (38.8)	9 (0.7)	3 (0.2)
Slightly severe	3874 (11.8)	1982 (51.2)	830 (21.4)	443 (11.4)	284 (7.3)	302 (7.8)	11 (0.3)	22 (0.6)
Moderately severe	10170 (30.9)	6160 (60.6)	2335 (23)	991 (9.7)	393 (3.9)	241 (2.4)	19 (0.2)	31 (0.3)
Severe	10373 (31.5)	7167 (69.1)	2108 (20.3)	634 (6.1)	200 (1.9)	135 (1.3)	22 (0.2)	107 (1)
Very severe	4586 (13.9)	3657 (79.7)	618 (13.5)	171 (3.7)	49 (1.1)	57 (1.2)	18 (0.4)	16 (0.3)
I don't know	2581 (7.8)	902 (34.9)	683 (26.5)	531 (20.6)	181 (7)	217 (8.4)	66 (2.6)	1 (0)
Missing	36 (0.1)	6 (16.7)	5 (13.9)	2 (5.6)	2 (5.6)	0 (0)	0 (0)	21 (58.3)
**Know someone or diagnosed with MPX**, N (%)								
No	26081 (79.3)	15085 (57.8)	5830 (22.4)	2550 (9.8)	1113 (4.3)	1295 (5)	98 (0.4)	110 (0.4)
Yes, only me	232 (0.7)	175 (75.4)	29 (12.5)	7 (3)	6 (2.6)	11 (4.7)	2 (0.9)	2 (0.9)
Yes, me and someone I know	619 (1.9)	495 (80)	66 (10.7)	23 (3.7)	9 (1.5)	19 (3.1)	4 (0.6)	3 (0.5)
Yes, only someone I know	3830 (11.6)	3180 (83)	365 (9.5)	117 (3.1)	43 (1.1)	59 (1.5)	8 (0.2)	58 (1.5)
I don't know	2018 (6.1)	1277 (63.3)	413 (20.5)	184 (9.1)	63 (3.1)	59 (2.9)	16 (0.8)	6 (0.3)
Prefer not to answer	83 (0.3)	43 (51.8)	10 (12)	6 (7.2)	2 (2.4)	5 (6)	17 (20.5)	0 (0)
Missing	39 (0.1)	11 (28.2)	1 (2.6)	3 (7.7)	0 (0)	2 (5.1)	0 (0)	22 (56.4)

Description of the sample using all vaccine acceptance question items. *(continued)*

Question	Sample Description	I will get vaccinated	Probably yes	Not sure	Probably not	I won't get vaccinated	Prefer not to answer	Missing
**Perception of vaccine protection in general**, N (%)								
Strongly disagree	1293 (4)	715 (55.3)	209 (16.2)	92 (7.1)	42 (3.2)	228 (17.6)	6 (0.5)	1 (0.1)
Slightly disagree	702 (2.2)	116 (16.5)	146 (20.8)	152 (21.7)	80 (11.4)	200 (28.5)	6 (0.9)	2 (0.3)
Neither disagree nor agree	951 (3)	112 (11.8)	156 (16.4)	249 (26.2)	159 (16.7)	247 (26)	26 (2.7)	2 (0.2)
Slightly agree	7139 (22.2)	3356 (47)	1777 (24.9)	1046 (14.7)	478 (6.7)	441 (6.2)	32 (0.4)	9 (0.1)
Strongly agree	21590 (67.3)	15414 (71.4)	4237 (19.6)	1212 (5.6)	414 (1.9)	263 (1.2)	37 (0.2)	13 (0.1)
I don't know	401 (1.2)	80 (20)	89 (22.2)	107 (26.7)	31 (7.7)	54 (13.5)	38 (9.5)	2 (0.5)
Missing	24 (0.1)	4 (16.7)	4 (16.7)	2 (8.3)	1 (4.2)	0 (0)	0 (0)	13 (54.2)
**Worried about being treated differently due to MPX**, N (%)								
No	12245 (38.1)	6640 (54.2)	2723 (22.2)	1228 (10)	658 (5.4)	940 (7.7)	51 (0.4)	5 (0)
Yes	11558 (36)	8273 (71.6)	2043 (17.7)	749 (6.5)	279 (2.4)	193 (1.7)	19 (0.2)	2 (0)
I don't know	5310 (16.5)	2858 (53.8)	1371 (25.8)	661 (12.4)	207 (3.9)	187 (3.5)	21 (0.4)	5 (0.1)
Prefer not to answer	257 (0.8)	82 (31.9)	50 (19.5)	38 (14.8)	17 (6.6)	21 (8.2)	49 (19.1)	0 (0)
Missing	2730 (8.5)	1944 (71.2)	431 (15.8)	184 (6.7)	44 (1.6)	92 (3.4)	5 (0.2)	30 (1.1)

S4. Description of the sample by region

The following tables describe the survey sample stratified by European subregion or residence of the respondents. See the manuscript for the description of the table.

Central Europe

Description of the sample for the Central Europe subregion.

Question	Sample Description	MPX Vaccine acceptance			
		Acceptance	Hesitancy	Refusal	No answer/Missing
**Total**, N (%)	2628 (100)	1760 (67)	376 (14.3)	472 (18)	20 (0.8)
**Preferred place to get vaccinated**, N (%)					
In an STI clinic	176 (7.4)	134 (76.1)	28 (15.9)	14 (8)	0 (0)
With my general practitioner	340 (14.3)	235 (69.1)	62 (18.2)	41 (12.1)	2 (0.6)
In a community-based centre	207 (8.7)	160 (77.3)	31 (15)	16 (7.7)	0 (0)
In a vaccination program centre	504 (21.2)	386 (76.6)	85 (16.9)	31 (6.2)	2 (0.4)
It doesn't matter	953 (40.2)	765 (80.3)	124 (13)	64 (6.7)	0 (0)
I don't know	180 (7.6)	76 (42.2)	46 (25.6)	45 (25)	13 (7.2)
Missing	12 (0.5)	4 (33.3)	0 (0)	5 (41.7)	3 (25)
**Age**, Median (IQR)	33.0 (26 - 41)	34.0 (26 - 42)	31.0 (24 - 38)	33.0 (26 - 41)	35.0 (26.75 - 41)
**Age category**, N (%)					
18-29	975 (37.1)	633 (64.9)	170 (17.4)	166 (17)	6 (0.6)
30-39	851 (32.4)	563 (66.2)	121 (14.2)	160 (18.8)	7 (0.8)
40-49	609 (23.2)	421 (69.1)	67 (11)	115 (18.9)	6 (1)
50-84	193 (7.3)	143 (74.1)	18 (9.3)	31 (16.1)	1 (0.5)
**Region of residence**, N (%)					
Central Europe	2628 (100)	1760 (67)	376 (14.3)	472 (18)	20 (0.8)
**Migrants**, N (%)					
No	2439 (92.8)	1641 (67.3)	350 (14.4)	432 (17.7)	16 (0.7)
Yes	189 (7.2)	119 (63)	26 (13.8)	40 (21.2)	4 (2.1)
**Migrant Origin**, N (%)					
Baltics	5 (2.6)	5 (100)	0 (0)	0 (0)	0 (0)
Central America	3 (1.6)	2 (66.7)	0 (0)	1 (33.3)	0 (0)
Central Europe	79 (41.8)	47 (59.5)	12 (15.2)	18 (22.8)	2 (2.5)
Eastern Europe	37 (19.6)	20 (54.1)	7 (18.9)	10 (27)	0 (0)
Eastern Mediterranean	4 (2.1)	4 (100)	0 (0)	0 (0)	0 (0)
Mediterranean Europe	11 (5.8)	7 (63.6)	2 (18.2)	2 (18.2)	0 (0)
Northern Europe	2 (1.1)	0 (0)	0 (0)	2 (100)	0 (0)
South America	5 (2.6)	5 (100)	0 (0)	0 (0)	0 (0)
South-East Asia	2 (1.1)	2 (100)	0 (0)	0 (0)	0 (0)
South-East Europe	13 (6.9)	8 (61.5)	1 (7.7)	3 (23.1)	1 (7.7)
Western Europe	23 (12.2)	16 (69.6)	3 (13)	4 (17.4)	0 (0)
Western Pacific	5 (2.6)	3 (60)	1 (20)	0 (0)	1 (20)
**HIV**, N (%)					
HIV-	2192 (83.4)	1456 (66.4)	324 (14.8)	399 (18.2)	13 (0.6)
HIV+ on ART	268 (10.2)	206 (76.9)	25 (9.3)	36 (13.4)	1 (0.4)
HIV+ not on ART	17 (0.6)	12 (70.6)	1 (5.9)	4 (23.5)	0 (0)

Description of the sample for the Central Europe subregion. *(continued)*

Question	Sample Description	Acceptance	Hesitancy	Refusal	No answer/Missing
HIV status unknown	115 (4.4)	64 (55.7)	22 (19.1)	26 (22.6)	3 (2.6)
Prefer not to answer	34 (1.3)	21 (61.8)	3 (8.8)	7 (20.6)	3 (8.8)
Missing	2 (0.1)	1 (50)	1 (50)	0 (0)	0 (0)
**PrEP**, N (%)					
PrEp no	2009 (85.7)	1277 (63.6)	320 (15.9)	397 (19.8)	15 (0.7)
PrEP yes	321 (13.7)	256 (79.8)	28 (8.7)	33 (10.3)	4 (1.2)
Missing	13 (0.6)	9 (69.2)	2 (15.4)	2 (15.4)	0 (0)
**STI in the last 12 months**, N (%)					
STI no	2251 (85.7)	1471 (65.3)	339 (15.1)	425 (18.9)	16 (0.7)
STI yes	266 (10.1)	212 (79.7)	23 (8.6)	31 (11.7)	0 (0)
STI unknown	36 (1.4)	23 (63.9)	5 (13.9)	7 (19.4)	1 (2.8)
Prefer not to answer	14 (0.5)	7 (50)	1 (7.1)	3 (21.4)	3 (21.4)
Missing	61 (2.3)	47 (77)	8 (13.1)	6 (9.8)	0 (0)
**Chemsex users**, N (%)					
Chemsex no	2340 (89)	1551 (66.3)	356 (15.2)	420 (17.9)	13 (0.6)
Chemsex yes	253 (9.6)	187 (73.9)	17 (6.7)	47 (18.6)	2 (0.8)
Prefer not to answer	32 (1.2)	20 (62.5)	3 (9.4)	4 (12.5)	5 (15.6)
Missing	3 (0.1)	2 (66.7)	0 (0)	1 (33.3)	0 (0)
**Perception of risk to get MPX**, N (%)					
Not worried	616 (23.4)	231 (37.5)	105 (17)	277 (45)	3 (0.5)
Slightly worried	732 (27.9)	518 (70.8)	120 (16.4)	90 (12.3)	4 (0.5)
Moderately worried	494 (18.8)	375 (75.9)	70 (14.2)	49 (9.9)	0 (0)
Worried	474 (18)	431 (90.9)	26 (5.5)	17 (3.6)	0 (0)
Very worried	172 (6.5)	152 (88.4)	8 (4.7)	11 (6.4)	1 (0.6)
I don't know	136 (5.2)	51 (37.5)	47 (34.6)	28 (20.6)	10 (7.4)
Missing	4 (0.2)	2 (50)	0 (0)	0 (0)	2 (50)
**Perception of MPX severity**, N (%)					
Not severe	168 (6.4)	33 (19.6)	18 (10.7)	114 (67.9)	3 (1.8)
Slightly severe	316 (12)	164 (51.9)	49 (15.5)	102 (32.3)	1 (0.3)
Moderately severe	770 (29.3)	545 (70.8)	122 (15.8)	102 (13.2)	1 (0.1)
Severe	769 (29.3)	640 (83.2)	83 (10.8)	45 (5.9)	1 (0.1)
Very severe	226 (8.6)	196 (86.7)	13 (5.8)	16 (7.1)	1 (0.4)
I don't know	374 (14.2)	180 (48.1)	91 (24.3)	92 (24.6)	11 (2.9)
Missing	5 (0.2)	2 (40)	0 (0)	1 (20)	2 (40)
**Know someone or diagnosed with MPX**, N (%)					
No	2357 (89.7)	1565 (66.4)	346 (14.7)	433 (18.4)	13 (0.6)
Yes, only me	13 (0.5)	6 (46.2)	2 (15.4)	5 (38.5)	0 (0)
Yes, me and someone I know	9 (0.3)	6 (66.7)	1 (11.1)	1 (11.1)	1 (11.1)
Yes, only someone I know	110 (4.2)	90 (81.8)	8 (7.3)	11 (10)	1 (0.9)
I don't know	131 (5)	93 (71)	17 (13)	21 (16)	0 (0)
Prefer not to answer	6 (0.2)	0 (0)	2 (33.3)	1 (16.7)	3 (50)
Missing	2 (0.1)	0 (0)	0 (0)	0 (0)	2 (100)
**Perception of vaccine protection in general**, N (%)					
Strongly disagree	222 (8.4)	136 (61.3)	14 (6.3)	71 (32)	1 (0.5)
Slightly disagree	108 (4.1)	24 (22.2)	23 (21.3)	60 (55.6)	1 (0.9)

Description of the sample for the Central Europe subregion. *(continued)*

Question	Sample Description	Acceptance	Hesitancy	Refusal	No answer/Missing
Neither disagree nor agree	132 (5)	22 (16.7)	39 (29.5)	68 (51.5)	3 (2.3)
Slightly agree	430 (16.4)	186 (43.3)	110 (25.6)	130 (30.2)	4 (0.9)
Strongly agree	1694 (64.5)	1383 (81.6)	180 (10.6)	127 (7.5)	4 (0.2)
I don't know	41 (1.6)	9 (22)	10 (24.4)	16 (39)	6 (14.6)
Missing	1 (0)	0 (0)	0 (0)	0 (0)	1 (100)
**Worried about being treated differently due to MPX**, N (%)					
No	1148 (43.7)	681 (59.3)	186 (16.2)	278 (24.2)	3 (0.3)
Yes	770 (29.3)	620 (80.5)	74 (9.6)	75 (9.7)	1 (0.1)
I don't know	621 (23.6)	438 (70.5)	109 (17.6)	71 (11.4)	3 (0.5)
Prefer not to answer	39 (1.5)	18 (46.2)	7 (17.9)	4 (10.3)	10 (25.6)
Missing	50 (1.9)	3 (6)	0 (0)	44 (88)	3 (6)

South-East Europe

Description of the sample for the South-East Europe subregion.

		MPX Vaccine acceptance			
Question	Sample Description	Acceptance	Hesitancy	Refusal	No answer/Missing
**Total**, N (%)	1901 (100)	1284 (67.5)	299 (15.7)	302 (15.9)	16 (0.8)
**Preferred place to get vaccinated**, N (%)					
In an STI clinic	219 (12.7)	167 (76.3)	42 (19.2)	8 (3.7)	2 (0.9)
With my general practitioner	214 (12.4)	147 (68.7)	50 (23.4)	15 (7)	2 (0.9)
In a community-based centre	132 (7.7)	100 (75.8)	22 (16.7)	10 (7.6)	0 (0)
In a vaccination program centre	351 (20.4)	253 (72.1)	71 (20.2)	26 (7.4)	1 (0.3)
It doesn't matter	674 (39.1)	579 (85.9)	69 (10.2)	24 (3.6)	2 (0.3)
I don't know	116 (6.7)	31 (26.7)	42 (36.2)	37 (31.9)	6 (5.2)
Missing	17 (1)	7 (41.2)	3 (17.6)	4 (23.5)	3 (17.6)
**Age**, Median (IQR)	33.0 (27 - 40)	34.0 (27 - 41)	32.0 (25 - 39)	32.0 (26 - 40)	31.5 (24.75 - 38)
**Age category**, N (%)					
18-29	686 (36.1)	438 (63.8)	126 (18.4)	115 (16.8)	7 (1)
30-39	679 (35.7)	469 (69.1)	102 (15)	103 (15.2)	5 (0.7)
40-49	396 (20.8)	277 (69.9)	55 (13.9)	63 (15.9)	1 (0.3)
50-84	140 (7.4)	100 (71.4)	16 (11.4)	21 (15)	3 (2.1)
**Region of residence**, N (%)					
South-East Europe	1901 (100)	1284 (67.5)	299 (15.7)	302 (15.9)	16 (0.8)
**Migrants**, N (%)					
No	1721 (90.5)	1159 (67.3)	278 (16.2)	269 (15.6)	15 (0.9)
Yes	180 (9.5)	125 (69.4)	21 (11.7)	33 (18.3)	1 (0.6)
**Migrant Origin**, N (%)					
Caribbean	1 (0.6)	1 (100)	0 (0)	0 (0)	0 (0)
Central Europe	6 (3.3)	2 (33.3)	1 (16.7)	3 (50)	0 (0)
Eastern Europe	15 (8.3)	11 (73.3)	1 (6.7)	3 (20)	0 (0)
Eastern Mediterranean	25 (13.9)	21 (84)	3 (12)	1 (4)	0 (0)

Description of the sample for the South-East Europe subregion. *(continued)*

Question	Sample Description	Acceptance	Hesitancy	Refusal	No answer/Missing
Mediterranean Europe	10 (5.6)	5 (50)	2 (20)	3 (30)	0 (0)
Northern America	7 (3.9)	7 (100)	0 (0)	0 (0)	0 (0)
South America	6 (3.3)	5 (83.3)	0 (0)	1 (16.7)	0 (0)
South-East Asia	2 (1.1)	1 (50)	1 (50)	0 (0)	0 (0)
South-East Europe	74 (41.1)	49 (66.2)	9 (12.2)	16 (21.6)	0 (0)
Sub-Saharan Africa	4 (2.2)	3 (75)	0 (0)	0 (0)	1 (25)
Western Europe	28 (15.6)	18 (64.3)	4 (14.3)	6 (21.4)	0 (0)
Western Pacific	2 (1.1)	2 (100)	0 (0)	0 (0)	0 (0)
**HIV**, N (%)					
HIV-	1478 (77.7)	977 (66.1)	242 (16.4)	249 (16.8)	10 (0.7)
HIV+ on ART	238 (12.5)	190 (79.8)	24 (10.1)	22 (9.2)	2 (0.8)
HIV+ not on ART	13 (0.7)	7 (53.8)	4 (30.8)	2 (15.4)	0 (0)
HIV status unknown	129 (6.8)	82 (63.6)	22 (17.1)	22 (17.1)	3 (2.3)
Prefer not to answer	32 (1.7)	21 (65.6)	7 (21.9)	4 (12.5)	0 (0)
Missing	11 (0.6)	7 (63.6)	0 (0)	3 (27.3)	1 (9.1)
**PrEP**, N (%)					
PrEp no	1421 (86.1)	926 (65.2)	246 (17.3)	240 (16.9)	9 (0.6)
PrEP yes	201 (12.2)	145 (72.1)	22 (10.9)	31 (15.4)	3 (1.5)
Missing	28 (1.7)	16 (57.1)	3 (10.7)	7 (25)	2 (7.1)
**STI in the last 12 months**, N (%)					
STI no	1565 (82.3)	1035 (66.1)	254 (16.2)	265 (16.9)	11 (0.7)
STI yes	260 (13.7)	201 (77.3)	34 (13.1)	23 (8.8)	2 (0.8)
STI unknown	60 (3.2)	36 (60)	9 (15)	13 (21.7)	2 (3.3)
Prefer not to answer	12 (0.6)	10 (83.3)	1 (8.3)	1 (8.3)	0 (0)
Missing	4 (0.2)	2 (50)	1 (25)	0 (0)	1 (25)
**Chemsex users**, N (%)					
Chemsex no	1681 (88.4)	1107 (65.9)	283 (16.8)	282 (16.8)	9 (0.5)
Chemsex yes	193 (10.2)	162 (83.9)	14 (7.3)	15 (7.8)	2 (1)
Prefer not to answer	26 (1.4)	15 (57.7)	2 (7.7)	5 (19.2)	4 (15.4)
Missing	1 (0.1)	0 (0)	0 (0)	0 (0)	1 (100)
**Perception of risk to get MPX**, N (%)					
Not worried	322 (16.9)	118 (36.6)	47 (14.6)	155 (48.1)	2 (0.6)
Slightly worried	488 (25.7)	321 (65.8)	95 (19.5)	71 (14.5)	1 (0.2)
Moderately worried	405 (21.3)	299 (73.8)	67 (16.5)	35 (8.6)	4 (1)
Worried	404 (21.3)	336 (83.2)	46 (11.4)	20 (5)	2 (0.5)
Very worried	200 (10.5)	170 (85)	20 (10)	10 (5)	0 (0)
I don't know	80 (4.2)	40 (50)	24 (30)	11 (13.8)	5 (6.2)
Missing	2 (0.1)	0 (0)	0 (0)	0 (0)	2 (100)
**Perception of MPX severity**, N (%)					
Not severe	97 (5.1)	22 (22.7)	20 (20.6)	55 (56.7)	0 (0)
Slightly severe	265 (13.9)	156 (58.9)	42 (15.8)	66 (24.9)	1 (0.4)
Moderately severe	592 (31.1)	421 (71.1)	96 (16.2)	71 (12)	4 (0.7)
Severe	324 (17)	255 (78.7)	36 (11.1)	31 (9.6)	2 (0.6)
Very severe	293 (15.4)	243 (82.9)	34 (11.6)	15 (5.1)	1 (0.3)
I don't know	328 (17.3)	187 (57)	71 (21.6)	64 (19.5)	6 (1.8)
Missing	2 (0.1)	0 (0)	0 (0)	0 (0)	2 (100)
**Know someone or diagnosed with MPX**, N (%)					
No	1686 (88.7)	1131 (67.1)	269 (16)	275 (16.3)	11 (0.7)

Description of the sample for the South-East Europe subregion. *(continued)*

Question	Sample Description	Acceptance	Hesitancy	Refusal	No answer/Missing
Yes, only me	7 (0.4)	5 (71.4)	0 (0)	1 (14.3)	1 (14.3)
Yes, me and someone I know	6 (0.3)	6 (100)	0 (0)	0 (0)	0 (0)
Yes, only someone I know	72 (3.8)	59 (81.9)	5 (6.9)	8 (11.1)	0 (0)
I don't know	121 (6.4)	81 (66.9)	23 (19)	17 (14)	0 (0)
Prefer not to answer	5 (0.3)	1 (20)	1 (20)	1 (20)	2 (40)
Missing	4 (0.2)	1 (25)	1 (25)	0 (0)	2 (50)
**Perception of vaccine protection in general**, N (%)					
Strongly disagree	269 (14.2)	191 (71)	26 (9.7)	51 (19)	1 (0.4)
Slightly disagree	129 (6.8)	49 (38)	34 (26.4)	46 (35.7)	0 (0)
Neither disagree nor agree	136 (7.2)	25 (18.4)	49 (36)	59 (43.4)	3 (2.2)
Slightly agree	417 (21.9)	210 (50.4)	105 (25.2)	97 (23.3)	5 (1.2)
Strongly agree	897 (47.2)	792 (88.3)	62 (6.9)	41 (4.6)	2 (0.2)
I don't know	50 (2.6)	17 (34)	21 (42)	8 (16)	4 (8)
Missing	3 (0.2)	0 (0)	2 (66.7)	0 (0)	1 (33.3)
**Worried about being treated differently due to MPX**, N (%)					
No	615 (32.4)	339 (55.1)	103 (16.7)	168 (27.3)	5 (0.8)
Yes	849 (44.7)	676 (79.6)	108 (12.7)	64 (7.5)	1 (0.1)
I don't know	409 (21.5)	258 (63.1)	84 (20.5)	64 (15.6)	3 (0.7)
Prefer not to answer	25 (1.3)	10 (40)	4 (16)	6 (24)	5 (20)
Missing	3 (0.2)	1 (33.3)	0 (0)	0 (0)	2 (66.7)

Baltics

Description of the sample for the Baltics subregion.

		MPX Vaccine acceptance		
Question	Sample Description	Acceptance	Hesitancy	Refusal
**Total**, N (%)	131 (100)	106 (80.9)	15 (11.5)	10 (7.6)
**Preferred place to get vaccinated**, N (%)				
In an STI clinic	10 (7.9)	9 (90)	1 (10)	0 (0)
With my general practitioner	16 (12.6)	13 (81.2)	3 (18.8)	0 (0)
In a community-based centre	3 (2.4)	3 (100)	0 (0)	0 (0)
In a vaccination program centre	23 (18.1)	21 (91.3)	1 (4.3)	1 (4.3)
It doesn't matter	69 (54.3)	59 (85.5)	6 (8.7)	4 (5.8)
I don't know	6 (4.7)	1 (16.7)	4 (66.7)	1 (16.7)
**Age**, Median (IQR)	34.0 (28.5 - 41)	34.0 (29 - 40)	32.0 (26.5 - 44.5)	35.0 (26.25 - 39.25)
**Age category**, N (%)				
18-29	38 (29)	30 (78.9)	5 (13.2)	3 (7.9)
30-39	56 (42.7)	47 (83.9)	5 (8.9)	4 (7.1)
40-49	25 (19.1)	20 (80)	3 (12)	2 (8)
50-84	12 (9.2)	9 (75)	2 (16.7)	1 (8.3)
**Region of residence**, N (%)				
Baltics	131 (100)	106 (80.9)	15 (11.5)	10 (7.6)

Description of the sample for the Baltics subregion. *(continued)*

Question	Sample Description	Acceptance	Hesitancy	Refusal
**Migrants**, N (%)				
No	97 (74)	76 (78.4)	13 (13.4)	8 (8.2)
Yes	34 (26)	30 (88.2)	2 (5.9)	2 (5.9)
**Migrant Origin**, N (%)				
Baltics	2 (5.9)	1 (50)	0 (0)	1 (50)
Central Europe	1 (2.9)	1 (100)	0 (0)	0 (0)
Eastern Europe	15 (44.1)	13 (86.7)	1 (6.7)	1 (6.7)
Eastern Mediterranean	1 (2.9)	1 (100)	0 (0)	0 (0)
Mediterranean Europe	4 (11.8)	4 (100)	0 (0)	0 (0)
Northern America	3 (8.8)	3 (100)	0 (0)	0 (0)
Northern Europe	4 (11.8)	3 (75)	1 (25)	0 (0)
Western Europe	2 (5.9)	2 (100)	0 (0)	0 (0)
Western Pacific	2 (5.9)	2 (100)	0 (0)	0 (0)
**HIV**, N (%)				
HIV-	116 (88.5)	98 (84.5)	9 (7.8)	9 (7.8)
HIV+ on ART	7 (5.3)	4 (57.1)	3 (42.9)	0 (0)
HIV+ not on ART	1 (0.8)	1 (100)	0 (0)	0 (0)
HIV status unknown	3 (2.3)	2 (66.7)	1 (33.3)	0 (0)
Prefer not to answer	2 (1.5)	1 (50)	1 (50)	0 (0)
Missing	2 (1.5)	0 (0)	1 (50)	1 (50)
**PrEP**, N (%)				
PrEp no	100 (81.3)	80 (80)	11 (11)	9 (9)
PrEP yes	20 (16.3)	20 (100)	0 (0)	0 (0)
Missing	3 (2.4)	1 (33.3)	1 (33.3)	1 (33.3)
**STI in the last 12 months**, N (%)				
STI no	114 (87)	91 (79.8)	14 (12.3)	9 (7.9)
STI yes	16 (12.2)	14 (87.5)	1 (6.2)	1 (6.2)
Missing	1 (0.8)	1 (100)	0 (0)	0 (0)
**Chemsex users**, N (%)				
Chemsex no	120 (91.6)	96 (80)	14 (11.7)	10 (8.3)
Chemsex yes	10 (7.6)	10 (100)	0 (0)	0 (0)
Prefer not to answer	1 (0.8)	0 (0)	1 (100)	0 (0)
**Perception of risk to get MPX**, N (%)				
Not worried	26 (19.8)	14 (53.8)	5 (19.2)	7 (26.9)
Slightly worried	40 (30.5)	33 (82.5)	5 (12.5)	2 (5)
Moderately worried	26 (19.8)	23 (88.5)	3 (11.5)	0 (0)
Worried	23 (17.6)	21 (91.3)	2 (8.7)	0 (0)
Very worried	15 (11.5)	14 (93.3)	0 (0)	1 (6.7)
I don't know	1 (0.8)	1 (100)	0 (0)	0 (0)
**Perception of MPX severity**, N (%)				
Not severe	5 (3.8)	2 (40)	1 (20)	2 (40)
Slightly severe	13 (9.9)	12 (92.3)	0 (0)	1 (7.7)
Moderately severe	48 (36.6)	40 (83.3)	5 (10.4)	3 (6.2)
Severe	37 (28.2)	33 (89.2)	3 (8.1)	1 (2.7)
Very severe	13 (9.9)	9 (69.2)	3 (23.1)	1 (7.7)
I don't know	14 (10.7)	9 (64.3)	3 (21.4)	2 (14.3)
Missing	1 (0.8)	1 (100)	0 (0)	0 (0)
**Know someone or diagnosed with MPX**, N (%)				
No	111 (84.7)	88 (79.3)	13 (11.7)	10 (9)
Yes, only me	1 (0.8)	1 (100)	0 (0)	0 (0)
Yes, me and someone I know	3 (2.3)	3 (100)	0 (0)	0 (0)
Yes, only someone I know	10 (7.6)	9 (90)	1 (10)	0 (0)
I don't know	6 (4.6)	5 (83.3)	1 (16.7)	0 (0)

Description of the sample for the Baltics subregion. *(continued)*

Question	Sample Description	Acceptance	Hesitancy	Refusal
**Perception of vaccine protection in general**, N (%)				
Strongly disagree	10 (7.6)	10 (100)	0 (0)	0 (0)
Slightly disagree	6 (4.6)	2 (33.3)	2 (33.3)	2 (33.3)
Neither disagree nor agree	3 (2.3)	2 (66.7)	1 (33.3)	0 (0)
Slightly agree	19 (14.5)	12 (63.2)	4 (21.1)	3 (15.8)
Strongly agree	92 (70.2)	80 (87)	7 (7.6)	5 (5.4)
I don't know	1 (0.8)	0 (0)	1 (100)	0 (0)
**Worried about being treated differently due to MPX**, N (%)				
No	41 (31.3)	29 (70.7)	10 (24.4)	2 (4.9)
Yes	58 (44.3)	52 (89.7)	2 (3.4)	4 (6.9)
I don't know	30 (22.9)	24 (80)	2 (6.7)	4 (13.3)
Missing	2 (1.5)	1 (50)	1 (50)	0 (0)

Eastern Europe

Description of the sample for the Eastern Europe subregion.

		MPX Vaccine acceptance			
**Question**	**Sample Description**	**Acceptance**	**Hesitancy**	**Refusal**	**No answer/Missing**
**Total**, N (%)	1602 (100)	1060 (66.2)	274 (17.1)	249 (15.5)	19 (1.2)
**Preferred place to get vaccinated**, N (%)					
In an STI clinic	66 (4.6)	51 (77.3)	12 (18.2)	3 (4.5)	0 (0)
With my general practitioner	181 (12.6)	117 (64.6)	46 (25.4)	15 (8.3)	3 (1.7)
In a community-based centre	98 (6.8)	78 (79.6)	14 (14.3)	6 (6.1)	0 (0)
In a vaccination program centre	356 (24.7)	264 (74.2)	70 (19.7)	19 (5.3)	3 (0.8)
It doesn't matter	667 (46.3)	524 (78.6)	109 (16.3)	32 (4.8)	2 (0.3)
I don't know	62 (4.3)	20 (32.3)	22 (35.5)	12 (19.4)	8 (12.9)
Missing	10 (0.7)	6 (60)	1 (10)	0 (0)	3 (30)
**Age**, Median (IQR)	31.0 (24 - 38)	31.0 (24 - 37)	31.0 (24 - 38)	34.0 (26 - 40)	33.0 (28.5 - 39)
**Age category**, N (%)					
18-29	695 (43.4)	473 (68.1)	120 (17.3)	96 (13.8)	6 (0.9)
30-39	589 (36.8)	387 (65.7)	105 (17.8)	89 (15.1)	8 (1.4)
40-49	255 (15.9)	167 (65.5)	35 (13.7)	49 (19.2)	4 (1.6)
50-84	63 (3.9)	33 (52.4)	14 (22.2)	15 (23.8)	1 (1.6)
**Region of residence**, N (%)					
Eastern Europe	1602 (100)	1060 (66.2)	274 (17.1)	249 (15.5)	19 (1.2)
**Migrants**, N (%)					
No	1422 (88.8)	940 (66.1)	243 (17.1)	225 (15.8)	14 (1)
Yes	180 (11.2)	120 (66.7)	31 (17.2)	24 (13.3)	5 (2.8)
**Migrant Origin**, N (%)					
Baltics	2 (1.1)	1 (50)	1 (50)	0 (0)	0 (0)
Caribbean	2 (1.1)	1 (50)	0 (0)	0 (0)	1 (50)
Central Europe	30 (16.7)	19 (63.3)	7 (23.3)	3 (10)	1 (3.3)

Description of the sample for the Eastern Europe subregion. *(continued)*

Question	Sample Description	Acceptance	Hesitancy	Refusal	No answer/Missing
Eastern Europe	119 (66.1)	83 (69.7)	18 (15.1)	15 (12.6)	3 (2.5)
Eastern Mediterranean	3 (1.7)	2 (66.7)	1 (33.3)	0 (0)	0 (0)
Mediterranean Europe	3 (1.7)	3 (100)	0 (0)	0 (0)	0 (0)
Northern America	2 (1.1)	1 (50)	1 (50)	0 (0)	0 (0)
South America	1 (0.6)	0 (0)	0 (0)	1 (100)	0 (0)
South-East Asia	3 (1.7)	2 (66.7)	1 (33.3)	0 (0)	0 (0)
South-East Europe	3 (1.7)	1 (33.3)	1 (33.3)	1 (33.3)	0 (0)
Sub-Saharan Africa	3 (1.7)	2 (66.7)	0 (0)	1 (33.3)	0 (0)
Western Europe	7 (3.9)	3 (42.9)	1 (14.3)	3 (42.9)	0 (0)
Western Pacific	2 (1.1)	2 (100)	0 (0)	0 (0)	0 (0)
**HIV**, N (%)					
HIV-	1236 (77.2)	818 (66.2)	208 (16.8)	198 (16)	12 (1)
HIV+ on ART	267 (16.7)	182 (68.2)	49 (18.4)	33 (12.4)	3 (1.1)
HIV+ not on ART	15 (0.9)	10 (66.7)	0 (0)	4 (26.7)	1 (6.7)
HIV status unknown	62 (3.9)	37 (59.7)	14 (22.6)	10 (16.1)	1 (1.6)
Prefer not to answer	18 (1.1)	11 (61.1)	2 (11.1)	4 (22.2)	1 (5.6)
Missing	4 (0.2)	2 (50)	1 (25)	0 (0)	1 (25)
**PrEP**, N (%)					
PrEp no	1168 (88.5)	757 (64.8)	204 (17.5)	196 (16.8)	11 (0.9)
PrEP yes	126 (9.5)	97 (77)	16 (12.7)	12 (9.5)	1 (0.8)
Missing	26 (2)	14 (53.8)	5 (19.2)	4 (15.4)	3 (11.5)
**STI in the last 12 months**, N (%)					
STI no	1380 (86.1)	903 (65.4)	242 (17.5)	222 (16.1)	13 (0.9)
STI yes	152 (9.5)	108 (71.1)	23 (15.1)	18 (11.8)	3 (2)
STI unknown	62 (3.9)	44 (71)	8 (12.9)	9 (14.5)	1 (1.6)
Prefer not to answer	4 (0.2)	2 (50)	1 (25)	0 (0)	1 (25)
Missing	4 (0.2)	3 (75)	0 (0)	0 (0)	1 (25)
**Chemsex users**, N (%)					
Chemsex no	1481 (92.4)	979 (66.1)	262 (17.7)	227 (15.3)	13 (0.9)
Chemsex yes	97 (6.1)	62 (63.9)	11 (11.3)	21 (21.6)	3 (3.1)
Prefer not to answer	21 (1.3)	17 (81)	1 (4.8)	1 (4.8)	2 (9.5)
Missing	3 (0.2)	2 (66.7)	0 (0)	0 (0)	1 (33.3)
**Perception of risk to get MPX**, N (%)					
Not worried	338 (21.1)	137 (40.5)	72 (21.3)	127 (37.6)	2 (0.6)
Slightly worried	439 (27.4)	301 (68.6)	84 (19.1)	51 (11.6)	3 (0.7)
Moderately worried	330 (20.6)	245 (74.2)	55 (16.7)	30 (9.1)	0 (0)
Worried	317 (19.8)	254 (80.1)	39 (12.3)	23 (7.3)	1 (0.3)
Very worried	124 (7.7)	102 (82.3)	12 (9.7)	8 (6.5)	2 (1.6)
I don't know	53 (3.3)	21 (39.6)	12 (22.6)	10 (18.9)	10 (18.9)
Missing	1 (0.1)	0 (0)	0 (0)	0 (0)	1 (100)
**Perception of MPX severity**, N (%)					
Not severe	63 (3.9)	18 (28.6)	5 (7.9)	40 (63.5)	0 (0)
Slightly severe	141 (8.8)	69 (48.9)	29 (20.6)	41 (29.1)	2 (1.4)
Moderately severe	489 (30.5)	316 (64.6)	97 (19.8)	74 (15.1)	2 (0.4)
Severe	433 (27)	332 (76.7)	59 (13.6)	40 (9.2)	2 (0.5)
Very severe	205 (12.8)	168 (82)	21 (10.2)	11 (5.4)	5 (2.4)
I don't know	269 (16.8)	157 (58.4)	62 (23)	43 (16)	7 (2.6)
Missing	2 (0.1)	0 (0)	1 (50)	0 (0)	1 (50)

Description of the sample for the Eastern Europe subregion. *(continued)*

Question	Sample Description	Acceptance	Hesitancy	Refusal	No answer/Missing
**Know someone or diagnosed with MPX**, N (%)					
No	1455 (90.8)	962 (66.1)	252 (17.3)	230 (15.8)	11 (0.8)
Yes, only me	1 (0.1)	1 (100)	0 (0)	0 (0)	0 (0)
Yes, me and someone I know	4 (0.2)	3 (75)	0 (0)	1 (25)	0 (0)
Yes, only someone I know	17 (1.1)	16 (94.1)	1 (5.9)	0 (0)	0 (0)
I don't know	119 (7.4)	78 (65.5)	21 (17.6)	17 (14.3)	3 (2.5)
Prefer not to answer	4 (0.2)	0 (0)	0 (0)	0 (0)	4 (100)
Missing	2 (0.1)	0 (0)	0 (0)	1 (50)	1 (50)
**Perception of vaccine protection in general**, N (%)					
Strongly disagree	56 (3.5)	18 (32.1)	4 (7.1)	32 (57.1)	2 (3.6)
Slightly disagree	118 (7.4)	36 (30.5)	38 (32.2)	43 (36.4)	1 (0.8)
Neither disagree nor agree	54 (3.4)	9 (16.7)	13 (24.1)	31 (57.4)	1 (1.9)
Slightly agree	289 (18)	144 (49.8)	79 (27.3)	64 (22.1)	2 (0.7)
Strongly agree	1018 (63.5)	831 (81.6)	121 (11.9)	59 (5.8)	7 (0.7)
I don't know	65 (4.1)	21 (32.3)	19 (29.2)	20 (30.8)	5 (7.7)
Missing	2 (0.1)	1 (50)	0 (0)	0 (0)	1 (50)
**Worried about being treated differently due to MPX**, N (%)					
No	754 (47.1)	448 (59.4)	131 (17.4)	169 (22.4)	6 (0.8)
Yes	361 (22.5)	273 (75.6)	53 (14.7)	35 (9.7)	0 (0)
I don't know	461 (28.8)	330 (71.6)	85 (18.4)	42 (9.1)	4 (0.9)
Prefer not to answer	22 (1.4)	8 (36.4)	4 (18.2)	3 (13.6)	7 (31.8)
Missing	4 (0.2)	1 (25)	1 (25)	0 (0)	2 (50)

Mediterranean Europe

Description of the sample for the Mediterranean Europe subregion.

		MPX Vaccine acceptance			
**Question**	**Sample Description**	**Acceptance**	**Hesitancy**	**Refusal**	**No answer/Missing**
**Total**, N (%)	11424 (100)	9826 (86)	856 (7.5)	686 (6)	56 (0.5)
**Preferred place to get vaccinated**, N (%)					
In an STI clinic	1241 (11.3)	1129 (91)	83 (6.7)	24 (1.9)	5 (0.4)
With my general practitioner	1811 (16.4)	1551 (85.6)	189 (10.4)	63 (3.5)	8 (0.4)
In a community-based centre	436 (4)	382 (87.6)	49 (11.2)	5 (1.1)	0 (0)
In a vaccination program centre	2262 (20.5)	1989 (87.9)	206 (9.1)	58 (2.6)	9 (0.4)
It doesn't matter	5057 (45.8)	4673 (92.4)	278 (5.5)	100 (2)	6 (0.1)
I don't know	186 (1.7)	82 (44.1)	48 (25.8)	38 (20.4)	18 (9.7)
Missing	38 (0.3)	20 (52.6)	3 (7.9)	5 (13.2)	10 (26.3)
**Age**, Median (IQR)	39.0 (31 - 47)	39.0 (31 - 47)	38.0 (29 - 48)	39.0 (30 - 49.75)	35.5 (28.75 - 43.25)

Description of the sample for the Mediterranean Europe subregion. *(continued)*

Question	Sample Description	Acceptance	Hesitancy	Refusal	No answer/Missing
**Age category**, N (%)					
18-29	2462 (21.6)	2070 (84.1)	221 (9)	154 (6.3)	17 (0.7)
30-39	3570 (31.2)	3097 (86.8)	248 (6.9)	204 (5.7)	21 (0.6)
40-49	3103 (27.2)	2735 (88.1)	204 (6.6)	156 (5)	8 (0.3)
50-84	2289 (20)	1924 (84.1)	183 (8)	172 (7.5)	10 (0.4)
**Region of residence**, N (%)					
Mediterranean Europe	11424 (100)	9826 (86)	856 (7.5)	686 (6)	56 (0.5)
**Migrants**, N (%)					
No	9505 (83.2)	8118 (85.4)	754 (7.9)	588 (6.2)	45 (0.5)
Yes	1919 (16.8)	1708 (89)	102 (5.3)	98 (5.1)	11 (0.6)
**Migrant Origin**, N (%)					
Baltics	4 (0.2)	4 (100)	0 (0)	0 (0)	0 (0)
Caribbean	51 (2.7)	45 (88.2)	3 (5.9)	2 (3.9)	1 (2)
Central America	92 (4.8)	89 (96.7)	0 (0)	3 (3.3)	0 (0)
Central Europe	73 (3.8)	53 (72.6)	16 (21.9)	4 (5.5)	0 (0)
Eastern Europe	27 (1.4)	20 (74.1)	3 (11.1)	4 (14.8)	0 (0)
Eastern Mediterranean	39 (2)	29 (74.4)	4 (10.3)	5 (12.8)	1 (2.6)
Mediterranean Europe	176 (9.2)	150 (85.2)	11 (6.2)	14 (8)	1 (0.6)
Northern America	50 (2.6)	43 (86)	3 (6)	4 (8)	0 (0)
Northern Europe	13 (0.7)	11 (84.6)	1 (7.7)	1 (7.7)	0 (0)
South America	972 (50.7)	908 (93.4)	39 (4)	22 (2.3)	3 (0.3)
South-East Asia	7 (0.4)	6 (85.7)	0 (0)	0 (0)	1 (14.3)
South-East Europe	38 (2)	28 (73.7)	1 (2.6)	8 (21.1)	1 (2.6)
Sub-Saharan Africa	67 (3.5)	55 (82.1)	5 (7.5)	5 (7.5)	2 (3)
Western Europe	288 (15)	246 (85.4)	15 (5.2)	26 (9)	1 (0.3)
Western Pacific	22 (1.1)	21 (95.5)	1 (4.5)	0 (0)	0 (0)
**HIV**, N (%)					
HIV-	9438 (82.6)	8075 (85.6)	738 (7.8)	583 (6.2)	42 (0.4)
HIV+ on ART	1404 (12.3)	1293 (92.1)	54 (3.8)	54 (3.8)	3 (0.2)
HIV+ not on ART	27 (0.2)	25 (92.6)	0 (0)	2 (7.4)	0 (0)
HIV status unknown	388 (3.4)	304 (78.4)	52 (13.4)	30 (7.7)	2 (0.5)
Prefer not to answer	131 (1.1)	101 (77.1)	8 (6.1)	14 (10.7)	8 (6.1)
Missing	36 (0.3)	28 (77.8)	4 (11.1)	3 (8.3)	1 (2.8)
**PrEP**, N (%)					
PrEp no	7918 (79.2)	6595 (83.3)	714 (9)	568 (7.2)	41 (0.5)
PrEP yes	1973 (19.7)	1834 (93)	75 (3.8)	54 (2.7)	10 (0.5)
Missing	102 (1)	79 (77.5)	13 (12.7)	8 (7.8)	2 (2)
**STI in the last 12 months**, N (%)					
STI no	9038 (79.1)	7663 (84.8)	715 (7.9)	616 (6.8)	44 (0.5)
STI yes	2179 (19.1)	2001 (91.8)	115 (5.3)	56 (2.6)	7 (0.3)
STI unknown	149 (1.3)	120 (80.5)	20 (13.4)	8 (5.4)	1 (0.7)
Prefer not to answer	49 (0.4)	36 (73.5)	6 (12.2)	5 (10.2)	2 (4.1)
Missing	9 (0.1)	6 (66.7)	0 (0)	1 (11.1)	2 (22.2)
**Chemsex users**, N (%)					
Chemsex no	10464 (91.6)	8957 (85.6)	822 (7.9)	637 (6.1)	48 (0.5)
Chemsex yes	871 (7.6)	798 (91.6)	32 (3.7)	38 (4.4)	3 (0.3)
Prefer not to answer	68 (0.6)	54 (79.4)	2 (2.9)	10 (14.7)	2 (2.9)
Missing	21 (0.2)	17 (81)	0 (0)	1 (4.8)	3 (14.3)

Description of the sample for the Mediterranean Europe subregion. *(continued)*

Question	Sample Description	Acceptance	Hesitancy	Refusal	No answer/Missing
**Perception of risk to get MPX**, N (%)					
Not worried	1173 (10.3)	628 (53.5)	153 (13)	385 (32.8)	7 (0.6)
Slightly worried	2627 (23)	2156 (82.1)	302 (11.5)	158 (6)	11 (0.4)
Moderately worried	2336 (20.4)	2099 (89.9)	170 (7.3)	58 (2.5)	9 (0.4)
Worried	2817 (24.7)	2615 (92.8)	141 (5)	52 (1.8)	9 (0.3)
Very worried	2303 (20.2)	2225 (96.6)	57 (2.5)	18 (0.8)	3 (0.1)
I don't know	158 (1.4)	98 (62)	33 (20.9)	15 (9.5)	12 (7.6)
Missing	10 (0.1)	5 (50)	0 (0)	0 (0)	5 (50)
**Perception of MPX severity**, N (%)					
Not severe	550 (4.8)	302 (54.9)	43 (7.8)	201 (36.5)	4 (0.7)
Slightly severe	1582 (13.8)	1287 (81.4)	149 (9.4)	140 (8.8)	6 (0.4)
Moderately severe	3567 (31.2)	3163 (88.7)	263 (7.4)	135 (3.8)	6 (0.2)
Severe	3171 (27.8)	2905 (91.6)	182 (5.7)	76 (2.4)	8 (0.3)
Very severe	1600 (14)	1515 (94.7)	54 (3.4)	24 (1.5)	7 (0.4)
I don't know	947 (8.3)	651 (68.7)	165 (17.4)	110 (11.6)	21 (2.2)
Missing	7 (0.1)	3 (42.9)	0 (0)	0 (0)	4 (57.1)
**Know someone or diagnosed with MPX**, N (%)					
No	9094 (79.6)	7691 (84.6)	746 (8.2)	619 (6.8)	38 (0.4)
Yes, only me	89 (0.8)	81 (91)	4 (4.5)	3 (3.4)	1 (1.1)
Yes, me and someone I know	322 (2.8)	295 (91.6)	14 (4.3)	12 (3.7)	1 (0.3)
Yes, only someone I know	1051 (9.2)	991 (94.3)	35 (3.3)	23 (2.2)	2 (0.2)
I don't know	804 (7)	716 (89.1)	55 (6.8)	26 (3.2)	7 (0.9)
Prefer not to answer	49 (0.4)	43 (87.8)	1 (2)	3 (6.1)	2 (4.1)
Missing	15 (0.1)	9 (60)	1 (6.7)	0 (0)	5 (33.3)
**Perception of vaccine protection in general**, N (%)					
Strongly disagree	368 (3.2)	294 (79.9)	23 (6.2)	50 (13.6)	1 (0.3)
Slightly disagree	161 (1.4)	77 (47.8)	28 (17.4)	54 (33.5)	2 (1.2)
Neither disagree nor agree	212 (1.9)	72 (34)	57 (26.9)	76 (35.8)	7 (3.3)
Slightly agree	2773 (24.3)	2181 (78.7)	298 (10.7)	279 (10.1)	15 (0.5)
Strongly agree	7779 (68.1)	7135 (91.7)	420 (5.4)	204 (2.6)	20 (0.3)
I don't know	128 (1.1)	65 (50.8)	30 (23.4)	22 (17.2)	11 (8.6)
Missing	3 (0)	2 (66.7)	0 (0)	1 (33.3)	0 (0)
**Worried about being treated differently due to MPX**, N (%)					
No	4104 (35.9)	3315 (80.8)	343 (8.4)	424 (10.3)	22 (0.5)
Yes	3346 (29.3)	3038 (90.8)	197 (5.9)	104 (3.1)	7 (0.2)
I don't know	1323 (11.6)	1137 (85.9)	120 (9.1)	61 (4.6)	5 (0.4)
Prefer not to answer	86 (0.8)	52 (60.5)	15 (17.4)	8 (9.3)	11 (12.8)
Missing	2565 (22.5)	2284 (89)	181 (7.1)	89 (3.5)	11 (0.4)

Northern Europe

Description of the sample for the Northern Europe subregion.

Question	Sample Description	MPX Vaccine acceptance			
		Acceptance	Hesitancy	Refusal	No answer/Missing
**Total**, N (%)	3126 (100)	2725 (87.2)	223 (7.1)	164 (5.2)	14 (0.4)
**Preferred place to get vaccinated**, N (%)					
In an STI clinic	429 (14)	400 (93.2)	22 (5.1)	7 (1.6)	0 (0)
With my general practitioner	322 (10.5)	265 (82.3)	49 (15.2)	8 (2.5)	0 (0)
In a community-based centre	98 (3.2)	88 (89.8)	9 (9.2)	1 (1)	0 (0)
In a vaccination program centre	428 (14)	369 (86.2)	39 (9.1)	20 (4.7)	0 (0)
It doesn't matter	1703 (55.6)	1572 (92.3)	78 (4.6)	51 (3)	2 (0.1)
I don't know	67 (2.2)	25 (37.3)	24 (35.8)	12 (17.9)	6 (9)
Missing	14 (0.5)	6 (42.9)	2 (14.3)	0 (0)	6 (42.9)
**Age**, Median (IQR)	40.0 (31 - 50)	41.0 (31 - 51)	38.0 (29 - 48)	37.5 (29 - 46)	36.0 (24 - 45.25)
**Age category**, N (%)					
18-29	672 (21.5)	559 (83.2)	64 (9.5)	43 (6.4)	6 (0.9)
30-39	813 (26)	706 (86.8)	56 (6.9)	50 (6.2)	1 (0.1)
40-49	810 (25.9)	713 (88)	53 (6.5)	38 (4.7)	6 (0.7)
50-84	831 (26.6)	747 (89.9)	50 (6)	33 (4)	1 (0.1)
**Region of residence**, N (%)					
Northern Europe	3126 (100)	2725 (87.2)	223 (7.1)	164 (5.2)	14 (0.4)
**Migrants**, N (%)					
No	2522 (80.7)	2197 (87.1)	182 (7.2)	136 (5.4)	7 (0.3)
Yes	604 (19.3)	528 (87.4)	41 (6.8)	28 (4.6)	7 (1.2)
**Migrant Origin**, N (%)					
Baltics	15 (2.5)	12 (80)	2 (13.3)	1 (6.7)	0 (0)
Caribbean	4 (0.7)	4 (100)	0 (0)	0 (0)	0 (0)
Central America	10 (1.7)	9 (90)	1 (10)	0 (0)	0 (0)
Central Europe	50 (8.3)	43 (86)	6 (12)	0 (0)	1 (2)
Eastern Europe	18 (3)	17 (94.4)	1 (5.6)	0 (0)	0 (0)
Eastern Mediterranean	42 (7)	34 (81)	3 (7.1)	2 (4.8)	3 (7.1)
Mediterranean Europe	70 (11.6)	62 (88.6)	2 (2.9)	5 (7.1)	1 (1.4)
Northern America	44 (7.3)	41 (93.2)	0 (0)	3 (6.8)	0 (0)
Northern Europe	78 (12.9)	69 (88.5)	7 (9)	2 (2.6)	0 (0)
South America	58 (9.6)	54 (93.1)	2 (3.4)	1 (1.7)	1 (1.7)
South-East Asia	20 (3.3)	14 (70)	4 (20)	2 (10)	0 (0)
South-East Europe	43 (7.1)	36 (83.7)	6 (14)	1 (2.3)	0 (0)
Sub-Saharan Africa	10 (1.7)	7 (70)	1 (10)	2 (20)	0 (0)
Western Europe	97 (16.1)	85 (87.6)	4 (4.1)	7 (7.2)	1 (1)
Western Pacific	45 (7.5)	41 (91.1)	2 (4.4)	2 (4.4)	0 (0)
**HIV**, N (%)					
HIV-	2787 (89.2)	2418 (86.8)	204 (7.3)	153 (5.5)	12 (0.4)
HIV+ on ART	252 (8.1)	238 (94.4)	9 (3.6)	5 (2)	0 (0)
HIV+ not on ART	10 (0.3)	6 (60)	2 (20)	2 (20)	0 (0)
HIV status unknown	49 (1.6)	39 (79.6)	6 (12.2)	3 (6.1)	1 (2)
Prefer not to answer	20 (0.6)	17 (85)	2 (10)	1 (5)	0 (0)
Missing	8 (0.3)	7 (87.5)	0 (0)	0 (0)	1 (12.5)
**PrEP**, N (%)					
PrEp no	2084 (72.8)	1734 (83.2)	198 (9.5)	141 (6.8)	11 (0.5)
PrEP yes	751 (26.2)	725 (96.5)	12 (1.6)	13 (1.7)	1 (0.1)

Description of the sample for the Northern Europe subregion. *(continued)*

Question	Sample Description	Acceptance	Hesitancy	Refusal	No answer/Missing
Missing	29 (1)	22 (75.9)	2 (6.9)	3 (10.3)	2 (6.9)
**STI in the last 12 months**, N (%)					
STI no	2506 (80.2)	2149 (85.8)	202 (8.1)	144 (5.7)	11 (0.4)
STI yes	577 (18.5)	544 (94.3)	14 (2.4)	18 (3.1)	1 (0.2)
STI unknown	33 (1.1)	24 (72.7)	7 (21.2)	2 (6.1)	0 (0)
Prefer not to answer	3 (0.1)	3 (100)	0 (0)	0 (0)	0 (0)
Missing	7 (0.2)	5 (71.4)	0 (0)	0 (0)	2 (28.6)
**Chemsex users**, N (%)					
Chemsex no	2927 (93.6)	2550 (87.1)	214 (7.3)	151 (5.2)	12 (0.4)
Chemsex yes	167 (5.3)	148 (88.6)	7 (4.2)	12 (7.2)	0 (0)
Prefer not to answer	25 (0.8)	22 (88)	2 (8)	1 (4)	0 (0)
Missing	7 (0.2)	5 (71.4)	0 (0)	0 (0)	2 (28.6)
**Perception of risk to get MPX**, N (%)					
Not worried	643 (20.6)	415 (64.5)	102 (15.9)	124 (19.3)	2 (0.3)
Slightly worried	927 (29.7)	838 (90.4)	63 (6.8)	25 (2.7)	1 (0.1)
Moderately worried	665 (21.3)	625 (94)	33 (5)	7 (1.1)	0 (0)
Worried	524 (16.8)	505 (96.4)	15 (2.9)	2 (0.4)	2 (0.4)
Very worried	315 (10.1)	307 (97.5)	3 (1)	5 (1.6)	0 (0)
I don't know	45 (1.4)	33 (73.3)	7 (15.6)	1 (2.2)	4 (8.9)
Missing	7 (0.2)	2 (28.6)	0 (0)	0 (0)	5 (71.4)
**Perception of MPX severity**, N (%)					
Not severe	106 (3.4)	58 (54.7)	7 (6.6)	41 (38.7)	0 (0)
Slightly severe	447 (14.3)	357 (79.9)	48 (10.7)	41 (9.2)	1 (0.2)
Moderately severe	1014 (32.4)	904 (89.2)	75 (7.4)	35 (3.5)	0 (0)
Severe	1011 (32.3)	929 (91.9)	51 (5)	27 (2.7)	4 (0.4)
Very severe	391 (12.5)	376 (96.2)	7 (1.8)	7 (1.8)	1 (0.3)
I don't know	150 (4.8)	98 (65.3)	35 (23.3)	13 (8.7)	4 (2.7)
Missing	7 (0.2)	3 (42.9)	0 (0)	0 (0)	4 (57.1)
**Know someone or diagnosed with MPX**, N (%)					
No	2517 (80.5)	2161 (85.9)	203 (8.1)	146 (5.8)	7 (0.3)
Yes, only me	9 (0.3)	7 (77.8)	0 (0)	2 (22.2)	0 (0)
Yes, me and someone I know	31 (1)	28 (90.3)	0 (0)	3 (9.7)	0 (0)
Yes, only someone I know	371 (11.9)	361 (97.3)	5 (1.3)	4 (1.1)	1 (0.3)
I don't know	188 (6)	164 (87.2)	15 (8)	8 (4.3)	1 (0.5)
Prefer not to answer	5 (0.2)	4 (80)	0 (0)	0 (0)	1 (20)
Missing	5 (0.2)	0 (0)	0 (0)	1 (20)	4 (80)
**Perception of vaccine protection in general**, N (%)					
Strongly disagree	93 (3)	78 (83.9)	6 (6.5)	9 (9.7)	0 (0)
Slightly disagree	38 (1.2)	19 (50)	3 (7.9)	15 (39.5)	1 (2.6)
Neither disagree nor agree	63 (2)	23 (36.5)	19 (30.2)	18 (28.6)	3 (4.8)
Slightly agree	399 (12.8)	282 (70.7)	59 (14.8)	55 (13.8)	3 (0.8)
Strongly agree	2506 (80.2)	2307 (92.1)	131 (5.2)	65 (2.6)	3 (0.1)
I don't know	23 (0.7)	15 (65.2)	5 (21.7)	2 (8.7)	1 (4.3)
Missing	4 (0.1)	1 (25)	0 (0)	0 (0)	3 (75)

Description of the sample for the Northern Europe subregion. *(continued)*

Question	Sample Description	Acceptance	Hesitancy	Refusal	No answer/Missing
**Worried about being treated differently due to MPX**, N (%)					
No	1357 (43.4)	1143 (84.2)	107 (7.9)	104 (7.7)	3 (0.2)
Yes	1209 (38.7)	1118 (92.5)	56 (4.6)	34 (2.8)	1 (0.1)
I don't know	529 (16.9)	443 (83.7)	58 (11)	25 (4.7)	3 (0.6)
Prefer not to answer	17 (0.5)	12 (70.6)	2 (11.8)	1 (5.9)	2 (11.8)
Missing	14 (0.4)	9 (64.3)	0 (0)	0 (0)	5 (35.7)

Western Europe

Description of the sample for the Western Europe subregion.

		MPX Vaccine acceptance			
**Question**	**Sample Description**	**Acceptance**	**Hesitancy**	**Refusal**	**No answer/Missing**
**Total**, N (%)	12090 (100)	10219 (84.5)	847 (7)	803 (6.6)	221 (1.8)
**Preferred place to get vaccinated**, N (%)					
In an STI clinic	1258 (11.5)	1122 (89.2)	93 (7.4)	40 (3.2)	3 (0.2)
With my general practitioner	2256 (20.7)	1950 (86.4)	210 (9.3)	91 (4)	5 (0.2)
In a community-based centre	260 (2.4)	227 (87.3)	28 (10.8)	2 (0.8)	3 (1.2)
In a vaccination program centre	1569 (14.4)	1363 (86.9)	143 (9.1)	60 (3.8)	3 (0.2)
It doesn't matter	5350 (49)	4914 (91.9)	286 (5.3)	141 (2.6)	9 (0.2)
I don't know	177 (1.6)	62 (35)	52 (29.4)	43 (24.3)	20 (11.3)
Missing	43 (0.4)	16 (37.2)	5 (11.6)	3 (7)	19 (44.2)
**Age**, Median (IQR)	41.0 (32 - 50)	42.0 (32 - 50)	39.0 (30 - 49)	39.0 (30 - 49)	41.0 (32 - 52)
**Age category**, N (%)					
18-29	2196 (18.2)	1764 (80.3)	209 (9.5)	190 (8.7)	33 (1.5)
30-39	3244 (26.8)	2734 (84.3)	222 (6.8)	217 (6.7)	71 (2.2)
40-49	3351 (27.7)	2887 (86.2)	209 (6.2)	210 (6.3)	45 (1.3)
50-84	3299 (27.3)	2834 (85.9)	207 (6.3)	186 (5.6)	72 (2.2)
**Region of residence**, N (%)					
Western Europe	12090 (100)	10219 (84.5)	847 (7)	803 (6.6)	221 (1.8)
**Migrants**, N (%)					
No	9818 (81.2)	8269 (84.2)	712 (7.3)	671 (6.8)	166 (1.7)
Yes	2272 (18.8)	1950 (85.8)	135 (5.9)	132 (5.8)	55 (2.4)
**Migrant Origin**, N (%)					
Baltics	19 (0.8)	16 (84.2)	0 (0)	2 (10.5)	1 (5.3)
Caribbean	28 (1.2)	24 (85.7)	2 (7.1)	0 (0)	2 (7.1)
Central America	45 (2)	41 (91.1)	1 (2.2)	2 (4.4)	1 (2.2)
Central Europe	181 (8)	147 (81.2)	11 (6.1)	18 (9.9)	5 (2.8)
Eastern Europe	61 (2.7)	45 (73.8)	7 (11.5)	8 (13.1)	1 (1.6)
Eastern Mediterranean	140 (6.2)	121 (86.4)	9 (6.4)	9 (6.4)	1 (0.7)
Mediterranean Europe	439 (19.3)	386 (87.9)	28 (6.4)	20 (4.6)	5 (1.1)
Northern America	104 (4.6)	94 (90.4)	4 (3.8)	6 (5.8)	0 (0)

Description of the sample for the Western Europe subregion. *(continued)*

Question	Sample Description	Acceptance	Hesitancy	Refusal	No answer/Missing
Northern Europe	33 (1.5)	32 (97)	0 (0)	1 (3)	0 (0)
South America	199 (8.8)	185 (93)	8 (4)	4 (2)	2 (1)
South-East Asia	33 (1.5)	27 (81.8)	3 (9.1)	2 (6.1)	1 (3)
South-East Europe	144 (6.3)	110 (76.4)	18 (12.5)	15 (10.4)	1 (0.7)
Sub-Saharan Africa	82 (3.6)	69 (84.1)	8 (9.8)	4 (4.9)	1 (1.2)
Western Europe	592 (26.1)	516 (87.2)	31 (5.2)	37 (6.2)	8 (1.4)
Western Pacific	77 (3.4)	71 (92.2)	3 (3.9)	1 (1.3)	2 (2.6)
Unclear Region	95 (4.2)	66 (69.5)	2 (2.1)	3 (3.2)	24 (25.3)
**HIV**, N (%)					
HIV-	10338 (85.5)	8696 (84.1)	742 (7.2)	712 (6.9)	188 (1.8)
HIV+ on ART	1344 (11.1)	1222 (90.9)	45 (3.3)	58 (4.3)	19 (1.4)
HIV+ not on ART	40 (0.3)	31 (77.5)	3 (7.5)	6 (15)	0 (0)
HIV status unknown	243 (2)	181 (74.5)	39 (16)	20 (8.2)	3 (1.2)
Prefer not to answer	102 (0.8)	70 (68.6)	17 (16.7)	7 (6.9)	8 (7.8)
Missing	23 (0.2)	19 (82.6)	1 (4.3)	0 (0)	3 (13)
**PrEP**, N (%)					
PrEp no	6826 (63.8)	5458 (80)	671 (9.8)	617 (9)	80 (1.2)
PrEP yes	3818 (35.7)	3466 (90.8)	122 (3.2)	118 (3.1)	112 (2.9)
Missing	62 (0.6)	42 (67.7)	6 (9.7)	4 (6.5)	10 (16.1)
**STI in the last 12 months**, N (%)					
STI no	9205 (76.1)	7645 (83.1)	727 (7.9)	709 (7.7)	124 (1.3)
STI yes	2706 (22.4)	2441 (90.2)	98 (3.6)	84 (3.1)	83 (3.1)
STI unknown	134 (1.1)	109 (81.3)	16 (11.9)	7 (5.2)	2 (1.5)
Prefer not to answer	29 (0.2)	16 (55.2)	5 (17.2)	3 (10.3)	5 (17.2)
Missing	16 (0.1)	8 (50)	1 (6.2)	0 (0)	7 (43.8)
**Chemsex users**, N (%)					
Chemsex no	10710 (88.6)	9030 (84.3)	799 (7.5)	738 (6.9)	143 (1.3)
Chemsex yes	1301 (10.8)	1136 (87.3)	42 (3.2)	62 (4.8)	61 (4.7)
Prefer not to answer	68 (0.6)	50 (73.5)	5 (7.4)	3 (4.4)	10 (14.7)
Missing	11 (0.1)	3 (27.3)	1 (9.1)	0 (0)	7 (63.6)
**Perception of risk to get MPX**, N (%)					
Not worried	1093 (9)	544 (49.8)	146 (13.4)	387 (35.4)	16 (1.5)
Slightly worried	2024 (16.7)	1529 (75.5)	238 (11.8)	216 (10.7)	41 (2)
Moderately worried	2976 (24.6)	2555 (85.9)	260 (8.7)	116 (3.9)	45 (1.5)
Worried	3710 (30.7)	3465 (93.4)	121 (3.3)	43 (1.2)	81 (2.2)
Very worried	2114 (17.5)	2034 (96.2)	43 (2)	25 (1.2)	12 (0.6)
I don't know	157 (1.3)	86 (54.8)	38 (24.2)	15 (9.6)	18 (11.5)
Missing	16 (0.1)	6 (37.5)	1 (6.2)	1 (6.2)	8 (50)
**Perception of MPX severity**, N (%)					
Not severe	293 (2.4)	92 (31.4)	24 (8.2)	172 (58.7)	5 (1.7)
Slightly severe	1110 (9.2)	767 (69.1)	126 (11.4)	195 (17.6)	22 (2)
Moderately severe	3690 (30.5)	3106 (84.2)	333 (9)	214 (5.8)	37 (1)
Severe	4628 (38.3)	4181 (90.3)	220 (4.8)	115 (2.5)	112 (2.4)
Very severe	1858 (15.4)	1768 (95.2)	39 (2.1)	32 (1.7)	19 (1)
I don't know	499 (4.1)	303 (60.7)	104 (20.8)	74 (14.8)	18 (3.6)
Missing	12 (0.1)	2 (16.7)	1 (8.3)	1 (8.3)	8 (66.7)
**Know someone or diagnosed with MPX**, N (%)					
No	8861 (73.3)	7317 (82.6)	721 (8.1)	695 (7.8)	128 (1.4)
Yes, only me	112 (0.9)	103 (92)	1 (0.9)	6 (5.4)	2 (1.8)

Description of the sample for the Western Europe subregion. *(continued)*

**Question**	**Sample Description**	**Acceptance**	**Hesitancy**	**Refusal**	**No answer/Missing**
Yes, me and someone I know	244 (2)	220 (90.2)	8 (3.3)	11 (4.5)	5 (2)
Yes, only someone I know	2199 (18.2)	2019 (91.8)	62 (2.8)	56 (2.5)	62 (2.8)
I don't know	649 (5.4)	553 (85.2)	52 (8)	33 (5.1)	11 (1.7)
Prefer not to answer	14 (0.1)	5 (35.7)	2 (14.3)	2 (14.3)	5 (35.7)
Missing	11 (0.1)	2 (18.2)	1 (9.1)	0 (0)	8 (72.7)
**Perception of vaccine protection in general**, N (%)					
Strongly disagree	275 (2.4)	197 (71.6)	19 (6.9)	57 (20.7)	2 (0.7)
Slightly disagree	142 (1.3)	55 (38.7)	24 (16.9)	60 (42.3)	3 (2.1)
Neither disagree nor agree	351 (3.1)	115 (32.8)	71 (20.2)	154 (43.9)	11 (3.1)
Slightly agree	2812 (24.9)	2118 (75.3)	391 (13.9)	291 (10.3)	12 (0.4)
Strongly agree	7604 (67.4)	7123 (93.7)	291 (3.8)	176 (2.3)	14 (0.2)
I don't know	93 (0.8)	42 (45.2)	21 (22.6)	17 (18.3)	13 (14)
Missing	11 (0.1)	4 (36.4)	0 (0)	0 (0)	7 (63.6)
**Worried about being treated differently due to MPX**, N (%)					
No	4226 (37.4)	3408 (80.6)	348 (8.2)	453 (10.7)	17 (0.4)
Yes	4965 (44)	4539 (91.4)	259 (5.2)	156 (3.1)	11 (0.2)
I don't know	1937 (17.2)	1599 (82.6)	203 (10.5)	127 (6.6)	8 (0.4)
Prefer not to answer	68 (0.6)	32 (47.1)	6 (8.8)	16 (23.5)	14 (20.6)
Missing	92 (0.8)	76 (82.6)	1 (1.1)	3 (3.3)	12 (13)

S5. Model specification

We investigated MPX vaccine by modelling the rate of respondents declaring to "surely" or "probably" willing to get vaccinated as a binomial process through a Bayesian multilevel logistic regression model. We developed two kind of models.
A random intercept model was used to evaluate baseline vaccine acceptance levels:

$$Pr(V_i) \sim \text{Binom}(p_i, N_i)$$

$$\begin{aligned}
\text{logit}(p_i) &= \beta_0 + u_{R_i} + u_{R_i*C_i} \\
u_{R_i} &\sim \text{Normal}(0, \sigma_R) \\
u_{R_i*C_i} &\sim \text{Normal}(0, \sigma_{R*C})
\end{aligned}$$

$$\begin{aligned}
\beta_0 &\sim \text{StudentT}(3, 0, 2.5) \\
\sigma_R &\sim \text{HalfNormal}(0, 1.5) \\
\sigma_{R*C} &\sim \text{HalfNormal}(0, 0.5)
\end{aligned}$$

with V_i being the number of respondents being sure or probably willing to vaccinate over the total N_i, in the group i identified by a country C_i and the respective European subregion of residence R_i. The group average vaccine acceptance is defined by p_i.
The term u_{R_i} and $u_{R_i*C_i}$ represent the deviates over the general mean β_0 (i.e., the model intercept) respectively due to the subregion and the country inside the suvregion of residence on the logit scale. These deviates are modelled as drawn by a normal distribution. The model priors are reported in the model formulation itself. The model was fitted with the R (R Core Team 2022) `brms` package (Bürkner 2017). The relative model formula is:

```
MPXWill | trials(N) ~ (1 | SubRegion/Country), family = binomial()
```

An expansion of this model with random slopes was used to assess the association of the investigated factors of interest with the vaccine acceptance rate. For each factor the following model was used:

$$Pr(V_{i,j}) \sim \text{Binom}(p_{i,j}, N_{i,j})$$

$$\begin{aligned}
\text{logit}(p_{i,j}) &= \beta_0 + u_{R_i} + u_{R_i*C_i} + X_j(\beta_j + \nu_{j,R_i} + \nu_{j,R_i*C_i}) \\
\begin{bmatrix} u_R \\ \nu_{1,R} \\ \cdots \\ \nu_{J,R} \end{bmatrix} &\sim \text{MVNormal}\Big(0, \mathbf{S_R}\Big), \begin{bmatrix} u_{R*C} \\ \nu_{1,R*C} \\ \cdots \\ \nu_{J,R*C} \end{bmatrix} \sim \text{MVNormal}\Big(0, \mathbf{S}_{J,R*C}\Big) \\
\mathbf{S}_R &= \begin{pmatrix} \sigma_R & & & \\ & \sigma_{1,R} & & \\ & & \ddots & \\ & & & \sigma_{J,R} \end{pmatrix} \mathbf{R}_R \begin{pmatrix} \sigma_R & & & \\ & \sigma_{1,R} & & \\ & & \ddots & \\ & & & \sigma_{J,R} \end{pmatrix} \\
\mathbf{S}_{R*C} &= \begin{pmatrix} \sigma_{R*C} & & & \\ & \sigma_{1,R*C} & & \\ & & \ddots & \\ & & & \sigma_{J,R*C} \end{pmatrix} \mathbf{R}_{R*C} \begin{pmatrix} \sigma_{R*C} & & & \\ & \sigma_{1,R*C} & & \\ & & \ddots & \\ & & & \sigma_{J,R*C} \end{pmatrix}
\end{aligned}$$

$$\begin{aligned}
\beta_0 &\sim \text{StudentT}(3, 0, 2.5) \\
\beta_{1\ldots J} &\sim \text{StudentT}(3, 0, 1.5) \\
(\sigma_R, \sigma_{1\ldots J,R}) &\sim \text{HalfNormal}(0, 1.5) \\
(\sigma_{R*C}, \sigma_{1\ldots J,R*C}) &\sim \text{HalfNormal}(0, 0.5) \\
(\mathbf{R}_R, \mathbf{R}_{R*C}) &\sim \text{LKJ}(2)
\end{aligned}$$

With β_j being the association parameter for the levels $j = 1 \ldots J$ of the factor of interest X (e.g., age groups, MPX severity perception, etc…), excluding a reference level incorporated in the intercept. In addition to the deviates on the intercept (u_{R_i} and $u_{R_i*C_i}$) we have also the deviates on the association parameter ν_{j,R_i} and ν_{j,R_i*C_i}. All the deviates at the same geographical level (SubRegion or Country inside SubRegion) are drawn from a multivariate normal distribution parameterised by the deviates marginal standard deviations σ and the correlation factor $\mathbf{R}$, which is give a restrictive LKJ(2) prior to reduce spurious correlation between the parameters. The relative model formula in the `brms` package is:

```
MPXWill | trials(N) ~ Pred + (Pred | SubRegion/Country), family = binomial()
```

with `Pred` being the factor of interest.

The models were fitted using 10,000 iterations on ten chains, with an adaptive delta of 0.99 (Carpenter et al. 2017). We checked model convergence through the $\hat{R}$ statistic and the number of divergent iterations, adapting priors in order to have the first always below 1.01 and the seconds close to zero.

For the baseline model we reported the median and (5%, 95%) quantiles (Credible Intervals, CrI) of the posterior distribution of the average vaccine acceptance p_i. To predict p_i for a given subregion we compute $\text{logit}^{-1}(\beta_0 + u_{R_i})$, while for each country we compute $\text{logit}^{-1}(\beta_0 + u_{R_i} + u_{R_i * C_i})$, for each set of posterior values of the parameters.

For the association models we reported the posterior distribution of the geographically adjusted relative risk (aRR) of vaccine acceptance, described as above (median and CrI), for a given factor value j compared to a reference value. We extracted the aRR from the association model analytically for each set of posterior values of the parameters:

$$aRR_j = \frac{\text{logit}^{-1}(\beta_0 + \beta_j)}{\text{logit}^{-1}(\beta_0)}$$

We also report the aRR for each subregion i and :

$$aRR_{i,j} = \frac{\text{logit}^{-1}(\beta_0 + u_{R_i} + \beta_{i,j} + \nu_{j,R_i})}{\text{logit}^{-1}(\beta_0 + u_{R_i})}$$

The subregion specific aRR are reported in the Supplemental Material S7.

For comparison reasons, we also computed the fixed effect version of both models, that is, omitting the geographical random effects.

For the posterior distribution of the aRR we also report two inferential statistics:

1. probability of direction (pD) which is defined as $Pr(aRR > 1)$ if the median aRR is > 1 and viceversa if lower. This value defines the probability of an association given the data (and the priors). A pD $> 95\%$ represent a strong evidence of effect (Makowski, Ben-Shachar, and Lüdecke 2019).
2. probability of the region of practical equivalence (pROPE), defined as $Pr(0.95 < aRR < 1.05)$. It represent the probability of a trivial, non relevant effect and is a practical proxy of the null effect probability ($Pr(aRR = 1)$) but has more preferable properties (Makowski, Ben-Shachar, and Lüdecke 2019). A pROPE $> 50\%$ indicate compelling evidence in favor of a low relevancy of the association, while above 90% we can solidy report a factor as non-relevant.

The union of these two statistics allows to clearly define the existence and the importance of an association, e.g., a high pD with a very high pROPE indicate a defined association but with no practical implication. A both low pD and low pROPE indicate that the data doesn't allow to draw any definite conclusion. A low pD but high pROPE is a strong evidence in favor of the null hypothesis, that is, absence of association.

S6. Baseline MPX vaccine acceptance probabilities.

We report here the estimated posterior vaccine acceptance. The estimates refer to the non-geographically adjusted model, to the adjusted model considering only the global intercept, and to the estimates at the regional and country levels. We highlighted respectively in red or green the estimates whose 50% CrI is lower or higher than the same interval at the level of the adjusted global estimates.

Baseline MPX vaccine acceptance probability, global and by European subregion and country.

Geographical Level	Location	Vaccine Acceptance	[90% CrI]	Sig. Diff.
**Global**	Global	77.8%	[68%, 85%]	average
	Northern Europe	87.8%	[84.8%, 90.4%]	higher
	Western Europe	85.6%	[83.1%, 87.7%]	higher
	Mediterranean Europe	84.7%	[81.4%, 87.3%]	higher
	Baltics	81.3%	[73.5%, 87.2%]	average
**SubRegion**	Central Europe	66.3%	[61.3%, 71%]	lower
	Eastern Europe	65.7%	[59.9%, 71.1%]	lower
	South-East Europe	65.7%	[60.9%, 70.2%]	lower
	Norway	91.5%	[88.5%, 93.9%]	higher
	Portugal	89.8%	[88.7%, 90.9%]	higher
	Denmark	89.7%	[87.8%, 91.4%]	higher
	Sweden	88.6%	[87.2%, 90%]	higher
	United Kingdom	88.6%	[85.5%, 91.2%]	higher
	Netherlands	87.5%	[85.5%, 89.4%]	higher
	Ireland	87.5%	[85.7%, 89.1%]	higher
	Spain	87.4%	[86.5%, 88.1%]	higher
	Belgium	87.3%	[85%, 89.3%]	higher
	Austria	86.9%	[84.8%, 88.9%]	higher
	Luxembourg	86.6%	[80.4%, 91.4%]	higher
	Iceland	86.6%	[81.2%, 90.7%]	higher
	France	86.3%	[85.5%, 87%]	higher
	Israel	85.5%	[83.6%, 87.4%]	higher
	Germany	85%	[83.9%, 86.1%]	higher
	Italy	85%	[84%, 86%]	higher
	Monaco	84.5%	[76.4%, 90%]	higher
	Andorra	83.5%	[75.9%, 89.1%]	higher
	Finland	83%	[80.8%, 85.1%]	higher
	Malta	83%	[77.7%, 87.3%]	higher
	Latvia	82.9%	[72.5%, 90%]	average

Baseline MPX vaccine acceptance probability, global and by European subregion and country. *(continued)*

Geographical Level	Location	Vaccine Acceptance	[90% CrI]	Sig. Diff.
Country	Estonia	82.5%	[75.1%, 88.4%]	average
	Switzerland	82.2%	[79.3%, 84.7%]	higher
	Liechtenstein	80.2%	[70.1%, 86.8%]	average
	Lithuania	79%	[71.3%, 85.3%]	average
	Croatia	76%	[72.4%, 79.4%]	average
	Greece	75.8%	[72.3%, 78.9%]	average
	Poland	72.2%	[69.8%, 74.5%]	lower
	Romania	71.9%	[68.4%, 75.2%]	lower
	Türkiye	70%	[67.3%, 72.7%]	lower
	Russia	68%	[65.8%, 70.1%]	lower
	Kyrgyzstan	67.1%	[55.4%, 77.3%]	lower
	Montenegro	67.1%	[56.5%, 76.4%]	lower
	Uzbekistan	67%	[56%, 76.7%]	lower
	Slovenia	67%	[55.4%, 77.1%]	lower
	Azerbaijan	66.5%	[55.3%, 76.3%]	lower
	Georgia	66.4%	[60.4%, 72.2%]	lower
	Armenia	66.4%	[54.9%, 76.5%]	lower
	Kazakhstan	65.9%	[58.5%, 72.5%]	lower
	Slovakia	65.1%	[58.3%, 71.3%]	lower
	Hungary	65%	[60.9%, 69.1%]	lower
	Ukraine	64.7%	[61.1%, 68.2%]	lower
	Republic of Moldova	64.6%	[52.8%, 75.2%]	lower
	Serbia	64.5%	[60.6%, 68.2%]	lower
	Albania	64.5%	[57.4%, 71.1%]	lower
	Tajikistan	62.6%	[49.1%, 73.6%]	lower
	North Macedonia	61.7%	[51.7%, 70.6%]	lower
	Cyprus	61%	[51.7%, 69.4%]	lower
	Bosnia and Herzegovina	60.2%	[49.8%, 69.5%]	lower
	Bulgaria	60%	[54.6%, 65.1%]	lower
	Belarus	57.8%	[50.3%, 64.9%]	lower
	Czechia	56.3%	[52%, 60.5%]	lower

Note:
Sig. Diff. represents a situation in which the 50% CrI is higher or lower than the global estimate 50% CrI.

S7. Factor association with MPX vaccine acceptance.

The following table report the estimated association of each factor investigated in the survey with the acceptance of the MPX vaccine. We report the location adjusted and unadjusted estimates.

Estimated factor association with MPX vaccine acceptance, unadjusted or adjusted by country of residence.

	unadjusted			adjusted		
**Question**	**RR [90% CrI]**	**pD**	**pROPE**	**RR [90% CrI]**	**pD**	**pROPE**
**Age category**						
30-39	1.06 [1.05, 1.07]	100%	14.4%	1.04 [1.01, 1.08]	97.2%	71.4%
40-49	1.09 [1.08, 1.1]	100%	0%	1.06 [1.02, 1.1]	99.3%	35.7%
50-84	1.1 [1.09, 1.12]	100%	0%	1.05 [1, 1.11]	94.2%	43.2%
**Migrants**						
Yes	1.05 [1.04, 1.06]	100%	34.1%	1.03 [1, 1.07]	93.9%	84.9%
**Migrant Origin**						
Western Pacific	1.13 [1.09, 1.17]	100%	0.2%	1.18 [1.07, 1.32]	99.1%	2.6%
Central America	1.15 [1.11, 1.18]	100%	0%	1.16 [0.98, 1.3]	94.1%	6.8%
Caribbean	1.11 [1.04, 1.16]	99.3%	7.3%	1.15 [0.98, 1.32]	93.4%	10.2%
South America	1.14 [1.12, 1.15]	100%	0%	1.15 [1.07, 1.27]	98.8%	2.4%
Northern America	1.09 [1.05, 1.13]	99.9%	5.3%	1.11 [0.99, 1.25]	94.2%	16.2%
Baltics	1.03 [0.92, 1.12]	69.4%	49.3%	1.07 [0.82, 1.24]	71.8%	25.7%
Mediterranean Europe	1.06 [1.04, 1.09]	100%	19.7%	1.05 [0.95, 1.15]	82.2%	47.6%
Eastern Mediterranean	1.04 [0.99, 1.08]	90.1%	69.6%	1.04 [0.92, 1.18]	74.1%	44.6%
Northern Europe	1.08 [1.01, 1.12]	97.8%	22.5%	1.02 [0.65, 1.19]	57.6%	29.1%
Western Europe	1.05 [1.03, 1.07]	100%	50.6%	1.02 [0.94, 1.08]	69.2%	73.7%
South-East Asia	0.98 [0.87, 1.06]	65.2%	58.6%	0.99 [0.74, 1.2]	54.4%	30.8%
Sub-Saharan Africa	1.02 [0.96, 1.08]	73.5%	75.9%	0.98 [0.78, 1.15]	59.5%	40.4%
Eastern Europe	0.89 [0.83, 0.94]	100%	1.8%	0.97 [0.86, 1.09]	66.2%	53.6%
Central Europe	0.93 [0.88, 0.97]	100%	16.3%	0.94 [0.82, 1.04]	85.3%	40.1%

Estimated factor association with MPX vaccine acceptance, unadjusted or adjusted by country of residence. *(continued)*

Question	RR [90% CrI]	pD	pROPE	RR [90% CrI]	pD	pROPE
South-East Europe	0.91 [0.86, 0.95]	100%	6.3%	0.91 [0.79, 1.01]	93.3%	24.5%
**HIV**						
HIV+ on ART	1.08 [1.07, 1.09]	100%	0%	1.1 [1.03, 1.17]	98.2%	8.1%
HIV+ not on ART	0.92 [0.84, 0.99]	97.3%	23.3%	0.96 [0.8, 1.1]	68.5%	43.2%
HIV status unknown	0.88 [0.85, 0.91]	100%	0%	0.9 [0.82, 0.96]	99.2%	7.6%
Prefer not to answer	0.92 [0.87, 0.97]	99.7%	15.8%	0.93 [0.83, 1.02]	89.4%	36.1%
Missing	0.99 [0.89, 1.06]	61.4%	65.7%	0.99 [0.71, 1.16]	55.1%	36%
**PrEP**						
PrEP yes	1.17 [1.16, 1.18]	100%	0%	1.19 [1.12, 1.28]	99.9%	0.2%
Missing	0.95 [0.89, 1.01]	91.6%	53.1%	0.96 [0.83, 1.06]	76.9%	47.8%
**STI in the last 12 months**						
STI yes	1.12 [1.11, 1.13]	100%	0%	1.14 [1.08, 1.21]	100%	0.4%
STI unknown	0.94 [0.9, 0.98]	99.7%	34.8%	0.96 [0.88, 1.03]	82%	60.1%
Prefer not to answer	0.92 [0.82, 1]	95.8%	25%	0.94 [0.75, 1.13]	71.5%	31.6%
Missing	1 [0.91, 1.07]	50.3%	68.8%	1.12 [0.98, 1.28]	92.9%	14.9%
**Chemsex users**						
Chemsex yes	1.08 [1.06, 1.09]	100%	0%	1.1 [1.03, 1.18]	98.9%	9.5%
Prefer not to answer	0.99 [0.94, 1.04]	59.7%	88.2%	1.04 [0.93, 1.14]	74.9%	52.6%
Missing	1.09 [0.97, 1.17]	90.7%	21.6%	1.14 [0.91, 1.32]	88.4%	13.2%
**Perception of risk to get MPX**						
Slightly worried	1.58 [1.53, 1.62]	100%	0%	1.69 [1.49, 1.96]	100%	0%
Moderately worried	1.73 [1.69, 1.78]	100%	0%	1.87 [1.6, 2.26]	100%	0%
Worried	1.86 [1.82, 1.91]	100%	0%	2.02 [1.69, 2.54]	100%	0%
Very worried	1.91 [1.86, 1.96]	100%	0%	2.06 [1.72, 2.61]	100%	0%
I don't know	1.15 [1.08, 1.23]	100%	0.9%	1.26 [1.11, 1.46]	99.2%	1.3%
Missing	1.7 [1.37, 1.92]	100%	0.1%	2.01 [1.5, 2.61]	99.6%	0.2%

Estimated factor association with MPX vaccine acceptance, unadjusted or adjusted by country of residence. *(continued)*

Question	RR [90% CrI]	pD	pROPE	RR [90% CrI]	pD	pROPE
**Perception of MPX severity**						
Slightly severe	1.75 [1.65, 1.85]	100%	0%	2.03 [1.71, 2.44]	100%	0%
Moderately severe	2.01 [1.9, 2.12]	100%	0%	2.42 [1.93, 3.12]	100%	0%
Severe	2.17 [2.05, 2.29]	100%	0%	2.65 [2.05, 3.58]	100%	0%
Very severe	2.25 [2.13, 2.38]	100%	0%	2.78 [2.12, 3.81]	100%	0%
I don't know	1.51 [1.42, 1.6]	100%	0%	1.82 [1.51, 2.25]	100%	0%
Missing	1.69 [1.19, 2.08]	98.8%	1%	2.15 [1.24, 3.2]	97.9%	0.7%
**Know someone or diagnosed with MPX**						
Yes, only me	1.11 [1.06, 1.14]	100%	2.4%	1.09 [0.91, 1.23]	84.8%	22.3%
Yes, me and someone I know	1.13 [1.11, 1.15]	100%	0%	1.14 [1.06, 1.26]	99.4%	2.7%
Yes, only someone I know	1.16 [1.15, 1.17]	100%	0%	1.2 [1.12, 1.31]	100%	0.1%
I don't know	1.05 [1.03, 1.06]	100%	60.7%	1.05 [1.01, 1.1]	97.8%	49.2%
Prefer not to answer	1 [0.89, 1.08]	53%	60.5%	0.8 [0.31, 1.12]	82.9%	13%
Missing	0.9 [0.67, 1.07]	80.7%	25.4%	0.73 [0.22, 1.1]	87.1%	10.8%
**Perception of vaccine protection in general**						
Slightly disagree	0.53 [0.48, 0.57]	100%	0%	0.53 [0.42, 0.65]	100%	0%
Neither disagree nor agree	0.41 [0.37, 0.44]	100%	0%	0.36 [0.27, 0.47]	100%	0%
Slightly agree	1.01 [0.98, 1.04]	66.3%	98.1%	0.83 [0.72, 0.93]	99.3%	2.6%
Strongly agree	1.27 [1.24, 1.31]	100%	0%	1.31 [1.19, 1.49]	100%	0%
I don't know	0.65 [0.59, 0.72]	100%	0%	0.63 [0.47, 0.79]	99.8%	0.3%
Missing	1.02 [0.71, 1.25]	55.4%	22.9%	1.15 [0.52, 1.49]	68.7%	10.1%

Estimated factor association with MPX vaccine acceptance, unadjusted or adjusted by country of residence. *(continued)*

Question	RR [90% CrI]	pD	pROPE	RR [90% CrI]	pD	pROPE
**Worried about being treated differently due to MPX**						
Yes	1.16 [1.15, 1.18]	100%	0%	1.23 [1.14, 1.35]	100%	0%
I don't know	1.04 [1.03, 1.06]	100%	82.9%	1.1 [1.04, 1.17]	99.8%	6.8%
Prefer not to answer	0.83 [0.76, 0.9]	100%	0.1%	0.84 [0.69, 0.98]	96.4%	8.1%
Missing	1.15 [1.13, 1.16]	100%	0%	1.07 [0.62, 1.33]	62.9%	17.2%

S8. Factor association with MPX vaccine acceptance by European subregion and country.

The following table report the estimated associations as in S7 but at the European subregion level. We highlighted respectively in red or green the estimates whose 50% CrI is lower or higher than the same interval at the level of the global estimates.

Estimated factor association with MPX vaccine acceptance at the regional level

Question	Value	Location	Estimate	[90% CrI]	pD	pROPE	Sig. Diff.
Age	30-39	Northern Europe	1.03	[1, 1.05]	96.4%	94%	average
		Mediterranean Europe	1.03	[1, 1.05]	96.2%	95.4%	average
		Baltics	1.04	[1, 1.09]	93.5%	69.8%	average
		Western Europe	1.04	[1.02, 1.07]	99.8%	73.9%	average
		Eastern Europe	1.04	[0.96, 1.1]	82.2%	58.3%	average
		Central Europe	1.04	[0.98, 1.09]	87.6%	58.5%	average
		South-East Europe	1.06	[1, 1.12]	95.4%	39%	average
	40-49	Northern Europe	1.04	[1.02, 1.06]	99.5%	79.5%	average
		Western Europe	1.04	[1.02, 1.07]	99.7%	65.1%	average
		Mediterranean Europe	1.05	[1.02, 1.07]	99.9%	62%	average
		Baltics	1.05	[1.01, 1.1]	96.1%	51.4%	average
		Eastern Europe	1.07	[0.99, 1.13]	92.2%	28.7%	average
		Central Europe	1.08	[1.02, 1.13]	97.7%	20.3%	average
		South-East Europe	1.08	[1.01, 1.14]	97%	19.6%	average
	50-84	Mediterranean Europe	1.03	[0.99, 1.07]	92.5%	78.4%	average
		Northern Europe	1.04	[1.01, 1.08]	98.5%	67.4%	average
		Western Europe	1.04	[1.01, 1.07]	97.7%	67.9%	average
		Baltics	1.04	[0.94, 1.11]	84.1%	53.3%	average
		Eastern Europe	1.05	[0.84, 1.14]	70%	33%	average
		South-East Europe	1.10	[1, 1.2]	95.7%	17.9%	average
		Central Europe	1.10	[1.02, 1.2]	97.3%	15.4%	average

	Chemsex yes	Northern Europe	1.03	[0.98, 1.07]	86.1%	74.8%	lower
		Eastern Europe	1.07	[0.87, 1.19]	74%	28.7%	average
		Western Europe	1.07	[1.04, 1.09]	100%	10.6%	average
		Mediterranean Europe	1.08	[1.05, 1.11]	100%	6.8%	average
		Baltics	1.10	[1.02, 1.21]	97.2%	13.3%	average
		Central Europe	1.14	[1.04, 1.22]	98.9%	5.7%	average
		South-East Europe	1.23	[1.14, 1.35]	100%	0.1%	higher
	Prefer not to answer	Mediterranean Europe	1.00	[0.9, 1.06]	50.7%	71.2%	average
		Baltics	1.01	[0.66, 1.12]	54.3%	46.5%	average
		Western Europe	1.02	[0.94, 1.07]	65.7%	74.6%	average
		Northern Europe	1.02	[0.93, 1.07]	65.7%	73.9%	average
		South-East Europe	1.05	[0.84, 1.23]	69.2%	30.6%	average
		Central Europe	1.09	[0.92, 1.26]	82.8%	26%	average
ChemSex		Eastern Europe	1.15	[0.97, 1.39]	90.9%	16.2%	average
	Missing	Western Europe	1.08	[0.8, 1.16]	75.8%	23.5%	average
		Northern Europe	1.09	[0.95, 1.15]	89%	22.9%	average
		Mediterranean Europe	1.11	[0.96, 1.18]	90.6%	17.7%	average
		Baltics	1.12	[0.73, 1.27]	80.7%	15.2%	average
		Central Europe	1.22	[0.71, 1.47]	80.4%	8.6%	average
		South-East Europe	1.25	[0.52, 1.53]	78.5%	7.9%	average
		Eastern Europe	1.29	[0.85, 1.53]	88.2%	6.9%	average
		Central Europe	0.43	[0.33, 0.54]	100%	0%	lower
		Eastern Europe	0.47	[0.37, 0.65]	100%	0%	average
		South-East Europe	0.48	[0.37, 0.58]	100%	0%	average
		Baltics	0.55	[0.39, 0.71]	99.9%	0.1%	average
		Mediterranean Europe	0.59	[0.48, 0.69]	100%	0%	average
		Western Europe	0.60	[0.49, 0.7]	100%	0%	average

**Slightly disagree**						
	Northern Europe	0.62	[0.49, 0.75]	100%	0%	average
**Neither disagree nor agree**	Central Europe	0.28	[0.21, 0.38]	100%	0%	lower
	Eastern Europe	0.29	[0.2, 0.41]	100%	0%	average
	South-East Europe	0.30	[0.2, 0.38]	100%	0%	average
	Baltics	0.40	[0.27, 0.61]	99.8%	0.1%	average
	Mediterranean Europe	0.42	[0.34, 0.53]	100%	0%	average
	Western Europe	0.43	[0.33, 0.52]	100%	0%	average
	Northern Europe	0.44	[0.32, 0.56]	100%	0%	average
**Slightly agree**	Central Europe	0.75	[0.6, 0.89]	99.6%	1.1%	average
	South-East Europe	0.77	[0.63, 0.89]	99.8%	0.6%	average
	Eastern Europe	0.79	[0.65, 0.97]	96.6%	5.1%	average
	Baltics	0.85	[0.69, 0.99]	96%	8.7%	average
	Mediterranean Europe	0.85	[0.75, 0.93]	99.8%	2.7%	average
	Northern Europe	0.87	[0.76, 0.96]	98.8%	6.4%	average
	Western Europe	0.92	[0.83, 1.03]	89.8%	28%	higher
**Strongly agree**	Northern Europe	1.20	[1.12, 1.31]	100%	0%	lower
	Western Europe	1.22	[1.15, 1.32]	100%	0%	lower
	Mediterranean Europe	1.23	[1.15, 1.33]	100%	0%	average
	Baltics	1.26	[1.12, 1.45]	99.8%	0.6%	average
	South-East Europe	1.40	[1.28, 1.59]	100%	0%	average
	Eastern Europe	1.50	[1.32, 1.77]	100%	0%	higher
	Central Europe	1.51	[1.34, 1.73]	100%	0%	higher
	Central Europe	0.48	[0.29, 0.65]	100%	0%	lower
	South-East Europe	0.53	[0.36, 0.69]	100%	0%	average
	Mediterranean Europe	0.62	[0.47, 0.75]	100%	0%	average
	Eastern Europe	0.62	[0.45, 0.87]	99%	1.5%	average

	**I don't know**	Baltics	0.63	[0.32, 0.88]	98.9%	1.5%	average
		Northern Europe	0.75	[0.58, 0.95]	97.9%	4%	average
		Western Europe	0.78	[0.65, 0.93]	99.4%	3%	higher
	**Missing**	South-East Europe	0.58	[0.026, 1.35]	76.3%	6.4%	average
		Mediterranean Europe	1.04	[0.49, 1.31]	56.2%	14.3%	average
		Baltics	1.12	[0.17, 1.48]	62.3%	8.5%	average
		Central Europe	1.14	[0.075, 1.87]	57.5%	5.4%	average
		Northern Europe	1.16	[0.62, 1.35]	74.5%	10.8%	average
		Western Europe	1.22	[0.88, 1.37]	89%	7.7%	average
		Eastern Europe	1.41	[0.51, 1.95]	77.7%	5.3%	average
	**HIV+ on ART**	Eastern Europe	1.05	[0.92, 1.17]	76.6%	40.1%	average
		Baltics	1.05	[0.83, 1.14]	73.8%	32.2%	average
		Northern Europe	1.07	[1.04, 1.11]	100%	9.8%	average
		Western Europe	1.08	[1.06, 1.1]	100%	1.7%	average
		Mediterranean Europe	1.09	[1.06, 1.12]	100%	0.4%	average
		Central Europe	1.15	[1.08, 1.23]	99.8%	1.7%	average
		South-East Europe	1.19	[1.1, 1.28]	99.9%	0.5%	higher
	**HIV+ not on ART**	South-East Europe	0.91	[0.63, 1.14]	73.1%	23.2%	average
		Northern Europe	0.91	[0.63, 1.02]	88.4%	33.6%	average
		Western Europe	0.94	[0.8, 1.03]	85.4%	42.6%	average
		Baltics	0.99	[0.73, 1.17]	54.4%	36.5%	average
		Eastern Europe	1.00	[0.76, 1.25]	50%	26.8%	average
		Central Europe	1.00	[0.78, 1.23]	50.3%	29.1%	average
		Mediterranean Europe	1.01	[0.9, 1.12]	56.4%	52.6%	average
		Central Europe	0.86	[0.77, 0.95]	99.4%	4.4%	average
		Eastern Europe	0.87	[0.77, 1]	95.3%	12.4%	average
		South-East Europe	0.89	[0.8, 1.01]	94.2%	17.2%	average

HIVStatus	HIV status unknown	Western Europe	0.91	[0.85, 0.95]	100%	5.2%	average
		Baltics	0.91	[0.79, 1]	95.1%	18.6%	average
		Mediterranean Europe	0.92	[0.87, 0.96]	100%	7.7%	average
		Northern Europe	0.94	[0.87, 0.99]	97.8%	30.5%	average
	Prefer not to answer	Eastern Europe	0.92	[0.75, 1.12]	77.7%	25.7%	average
		Western Europe	0.92	[0.83, 0.99]	98.1%	26.1%	average
		South-East Europe	0.93	[0.78, 1.1]	78.3%	28.1%	average
		Central Europe	0.93	[0.79, 1.11]	76.2%	30.4%	average
		Baltics	0.94	[0.75, 1.05]	84%	36.5%	average
		Mediterranean Europe	0.95	[0.89, 1.01]	89.9%	52.5%	average
		Northern Europe	0.96	[0.87, 1.03]	84.9%	57.4%	average
	Missing	Baltics	0.83	[0.13, 1.08]	80.8%	20.9%	average
		Central Europe	0.93	[0.36, 1.29]	62.3%	17.1%	average
		Mediterranean Europe	0.96	[0.79, 1.07]	70.6%	46.5%	average
		Eastern Europe	0.96	[0.45, 1.33]	56.9%	17.8%	average
		South-East Europe	1.02	[0.7, 1.31]	54.7%	22%	average
		Northern Europe	1.04	[0.89, 1.14]	71.1%	40.2%	average
		Western Europe	1.05	[0.92, 1.15]	74.8%	39%	average
	Yes, only me	Central Europe	0.92	[0.52, 1.21]	63.1%	16.9%	average
		Northern Europe	1.03	[0.8, 1.11]	62.1%	40.6%	average
		Mediterranean Europe	1.08	[1, 1.14]	94.8%	23%	average
		Baltics	1.09	[0.78, 1.25]	78.1%	19.8%	average
		Western Europe	1.10	[1.04, 1.16]	99.4%	6.9%	average
		Eastern Europe	1.17	[0.69, 1.47]	77.6%	10.9%	average
		South-East Europe	1.18	[0.84, 1.44]	83.4%	11.7%	average
		Northern Europe	1.07	[1, 1.12]	95.1%	21.6%	lower
		Mediterranean Europe	1.09	[1.05, 1.13]	99.6%	6.7%	lower

**Yes, me and someone I know**	Western Europe	1.10	[1.06, 1.14]	100%	1.6%	average
	Baltics	1.12	[1.03, 1.23]	96.8%	7.4%	average
	Central Europe	1.22	[1.02, 1.38]	95.8%	4.3%	average
	Eastern Europe	1.23	[1.02, 1.4]	95.7%	3.9%	average
	South-East Europe	1.25	[1.09, 1.46]	98.4%	2.2%	higher
**Yes, only someone I know**	Northern Europe	1.12	[1.09, 1.15]	100%	0%	lower
	Western Europe	1.13	[1.1, 1.15]	100%	0%	lower
	Mediterranean Europe	1.13	[1.1, 1.16]	100%	0%	lower
	Baltics	1.16	[1.09, 1.25]	99.6%	1.1%	average
	South-East Europe	1.30	[1.19, 1.39]	100%	0.1%	higher
	Central Europe	1.30	[1.22, 1.39]	100%	0%	higher
	Eastern Europe	1.32	[1.23, 1.45]	100%	0.1%	higher
**I don't know**	Northern Europe	1.03	[1, 1.06]	94.3%	88.3%	average
	Western Europe	1.04	[1.01, 1.06]	98.1%	80.7%	average
	Baltics	1.04	[0.99, 1.1]	91.9%	58.3%	average
	Mediterranean Europe	1.04	[1.02, 1.08]	99.5%	64.4%	average
	South-East Europe	1.07	[0.98, 1.14]	89.9%	32.9%	average
	Eastern Europe	1.07	[0.97, 1.14]	89.9%	32.1%	average
	Central Europe	1.07	[0.99, 1.15]	94.1%	27.1%	average
**Prefer not to answer**	Central Europe	0.33	[0.018, 0.94]	96.6%	2.6%	lower
	South-East Europe	0.58	[0.12, 1.12]	89.2%	5.6%	average
	Eastern Europe	0.67	[0.046, 1.42]	75.2%	6.3%	average
	Western Europe	0.77	[0.42, 1.01]	94.1%	10.7%	average
	Baltics	0.85	[0.11, 1.21]	70%	13.1%	average
	Northern Europe	1.03	[0.73, 1.16]	58.3%	26.1%	average
	Mediterranean Europe	1.03	[0.82, 1.13]	62.4%	37.1%	higher
	Eastern Europe	0.57	[0.042, 1.17]	87.8%	5.9%	average

	Missing	Central Europe	0.66	[0.053, 1.35]	78.6%	6.7%	average
		South-East Europe	0.67	[0.16, 1.24]	82%	7.3%	average
		Northern Europe	0.73	[0.073, 1.05]	89.6%	12.7%	average
		Baltics	0.78	[0.1, 1.17]	78.2%	11.9%	average
		Western Europe	0.83	[0.39, 1.1]	82.5%	17%	average
		Mediterranean Europe	0.95	[0.64, 1.14]	64.6%	25.8%	average
Migrant	Yes	Northern Europe	1.01	[0.99, 1.03]	84.9%	99.3%	average
		Western Europe	1.02	[1, 1.04]	96.6%	97.7%	average
		Mediterranean Europe	1.02	[1, 1.05]	96%	95.3%	average
		Baltics	1.03	[0.99, 1.08]	90.8%	82.4%	average
		Central Europe	1.04	[0.96, 1.09]	82.2%	60.6%	average
		Eastern Europe	1.04	[0.97, 1.1]	86.3%	55.3%	average
		South-East Europe	1.04	[0.97, 1.1]	86.8%	55.7%	average
	Baltics	Northern Europe	1.00	[0.82, 1.09]	51.8%	50.6%	average
		Baltics	1.00	[0.5, 1.18]	51%	25.5%	average
		Western Europe	1.04	[0.89, 1.13]	71.4%	40.2%	average
		Eastern Europe	1.06	[0.49, 1.39]	59.5%	14.6%	average
		Mediterranean Europe	1.07	[0.87, 1.18]	77%	27.7%	average
		South-East Europe	1.11	[0.44, 1.47]	65.7%	11.6%	average
		Central Europe	1.24	[0.88, 1.5]	86.4%	9.8%	average
		Northern Europe	1.08	[0.98, 1.14]	91.8%	22.4%	average
		Mediterranean Europe	1.09	[0.98, 1.15]	91.2%	23.4%	average
		Western Europe	1.09	[0.99, 1.15]	94.1%	18.8%	average
		Baltics	1.12	[0.86, 1.26]	86.5%	13.1%	average
		Central Europe	1.25	[0.75, 1.5]	86.4%	6.8%	average
		Eastern Europe	1.27	[0.88, 1.52]	89.6%	6.7%	average

**Caribbean**						
	South-East Europe	1.28	[0.89, 1.52]	90%	6.3%	average
**Central America**	Northern Europe	1.08	[0.95, 1.13]	89.3%	22.1%	lower
	Western Europe	1.10	[1.03, 1.15]	97.9%	9.9%	average
	Mediterranean Europe	1.13	[1.06, 1.18]	99.6%	2.7%	average
	Baltics	1.13	[0.8, 1.26]	87%	10.1%	average
	Central Europe	1.25	[0.79, 1.44]	86.6%	6.6%	average
	Eastern Europe	1.27	[0.68, 1.49]	86.7%	4.6%	average
	South-East Europe	1.28	[0.7, 1.49]	87%	5%	average
**Central Europe**	South-East Europe	0.87	[0.5, 1.06]	87.7%	17.6%	average
	Mediterranean Europe	0.92	[0.78, 1]	95%	28.8%	average
	Eastern Europe	0.93	[0.75, 1.14]	72.2%	28.1%	average
	Central Europe	0.94	[0.8, 1.09]	75%	34.5%	average
	Baltics	0.96	[0.76, 1.11]	72.7%	41.2%	average
	Western Europe	0.97	[0.91, 1.03]	79.1%	69.3%	average
	Northern Europe	0.97	[0.9, 1.04]	74%	69.6%	average
**Eastern Europe**	Central Europe	0.90	[0.68, 1.06]	84.7%	24.9%	average
	Mediterranean Europe	0.94	[0.76, 1.03]	84.7%	43%	average
	Western Europe	0.95	[0.82, 1.02]	86.6%	45.7%	average
	Northern Europe	1.00	[0.92, 1.09]	51.9%	68.7%	average
	Baltics	1.00	[0.88, 1.15]	51.8%	49.8%	average
	South-East Europe	1.01	[0.81, 1.25]	52.4%	30.7%	average
	Eastern Europe	1.02	[0.86, 1.17]	56.7%	41.3%	average
	Mediterranean Europe	0.98	[0.82, 1.06]	64.6%	54.9%	average
	Northern Europe	1.01	[0.92, 1.08]	60.6%	72.2%	average
	Western Europe	1.02	[0.95, 1.07]	69.3%	76.6%	average
	Baltics	1.04	[0.83, 1.2]	68.5%	38.6%	average
	Eastern Europe	1.06	[0.72, 1.35]	66%	23.1%	average

**Eastern Mediterranean**						
	Central Europe	1.13	[0.88, 1.44]	81.1%	19.3%	average
	South-East Europe	1.13	[0.93, 1.36]	85.5%	18.6%	average
**Mediterranean Europe**	Mediterranean Europe	1.02	[0.94, 1.08]	69.7%	72.5%	average
	Northern Europe	1.03	[0.96, 1.08]	80.8%	75.1%	average
	South-East Europe	1.03	[0.7, 1.2]	59.8%	28%	average
	Western Europe	1.04	[1, 1.09]	95.6%	59.4%	average
	Baltics	1.05	[0.92, 1.18]	81.4%	43.3%	average
	Central Europe	1.06	[0.8, 1.23]	69.8%	28.3%	average
	Eastern Europe	1.10	[0.89, 1.36]	82.2%	21.6%	average
**Northern America**	Mediterranean Europe	1.05	[0.95, 1.11]	82.5%	45.5%	average
	Northern Europe	1.05	[0.99, 1.11]	92.3%	43.8%	average
	Western Europe	1.06	[1, 1.11]	95.8%	34%	average
	Baltics	1.09	[0.95, 1.24]	89.8%	19.6%	average
	Eastern Europe	1.14	[0.7, 1.38]	79.5%	12%	average
	Central Europe	1.17	[0.75, 1.43]	84.6%	10.3%	average
	South-East Europe	1.24	[1.03, 1.5]	96.4%	5.5%	average
**Northern Europe**	Central Europe	0.81	[0.087, 1.24]	69.5%	12.8%	average
	Baltics	1.01	[0.62, 1.18]	53.7%	28.7%	average
	Northern Europe	1.01	[0.91, 1.08]	61.4%	67.3%	average
	South-East Europe	1.03	[0.19, 1.44]	54.2%	12.5%	average
	Mediterranean Europe	1.03	[0.83, 1.14]	64.7%	39%	average
	Eastern Europe	1.03	[0.21, 1.44]	55%	12.9%	average
	Western Europe	1.09	[0.99, 1.16]	92.6%	23.2%	average
	Northern Europe	1.08	[1.03, 1.12]	98.8%	11.4%	lower
	Western Europe	1.10	[1.07, 1.14]	100%	1.2%	lower
	Mediterranean Europe	1.10	[1.07, 1.14]	100%	0.5%	lower

South America	Baltics	1.12	[0.99, 1.23]	94.5%	6.4%	average
	Eastern Europe	1.24	[0.79, 1.39]	89.4%	4.6%	average
	South-East Europe	1.26	[1.05, 1.42]	96.7%	2.8%	higher
	Central Europe	1.27	[1.12, 1.45]	98.8%	1.5%	higher
South-East Asia	Northern Europe	0.91	[0.68, 1.04]	86.1%	30.8%	average
	South-East Europe	0.92	[0.4, 1.33]	62%	15.2%	average
	Eastern Europe	0.98	[0.54, 1.37]	54.3%	16.1%	average
	Baltics	0.98	[0.49, 1.2]	56.2%	25.6%	average
	Western Europe	0.99	[0.85, 1.09]	57.2%	52%	average
	Mediterranean Europe	1.03	[0.84, 1.17]	60.2%	33.1%	average
	Central Europe	1.08	[0.68, 1.47]	61.3%	14.5%	average
South-East Europe	Eastern Europe	0.86	[0.58, 1.06]	89.7%	13.5%	average
	Central Europe	0.91	[0.74, 1.14]	77.8%	21%	average
	South-East Europe	0.91	[0.78, 1.06]	83.3%	26.4%	average
	Western Europe	0.92	[0.85, 0.98]	99%	21.3%	average
	Baltics	0.93	[0.73, 1.06]	85.5%	30.2%	average
	Mediterranean Europe	0.93	[0.82, 1]	94.4%	30.4%	average
	Northern Europe	0.95	[0.86, 1.01]	91.6%	47%	average
Sub-Saharan Africa	Northern Europe	0.96	[0.72, 1.05]	76.3%	49.3%	average
	Central Europe	0.97	[0.5, 1.36]	55.9%	20.5%	average
	Eastern Europe	0.97	[0.61, 1.31]	55.8%	21.9%	average
	Mediterranean Europe	0.97	[0.87, 1.06]	67.6%	58.3%	average
	Baltics	0.98	[0.62, 1.17]	58.8%	36.5%	average
	Western Europe	0.98	[0.89, 1.06]	63.4%	67%	average
	South-East Europe	1.04	[0.74, 1.43]	57.5%	20.5%	average
	Northern Europe	1.01	[0.96, 1.05]	66.8%	92.1%	average
	Eastern Europe	1.01	[0.75, 1.13]	55.4%	42.1%	average

		Mediterranean Europe	1.01	[0.96, 1.05]	67.1%	89.8%	average
		Baltics	1.02	[0.91, 1.11]	68.3%	66.2%	average
	**Western Europe**	South-East Europe	1.02	[0.85, 1.14]	60.8%	45.7%	average
		Western Europe	1.02	[0.99, 1.06]	86.2%	89.9%	average
		Central Europe	1.04	[0.89, 1.16]	69.3%	45.1%	average
		Northern Europe	1.08	[1.01, 1.13]	96.5%	18.2%	lower
		Western Europe	1.11	[1.06, 1.16]	99.8%	3.3%	lower
		Mediterranean Europe	1.12	[1.05, 1.18]	98.7%	5.3%	average
	**Western Pacific**	Baltics	1.15	[1.02, 1.27]	96.3%	6.3%	average
		Central Europe	1.29	[1.02, 1.46]	95.5%	3.4%	average
		Eastern Europe	1.32	[1.07, 1.52]	97.2%	2.3%	higher
		South-East Europe	1.32	[1.07, 1.5]	97.2%	2.2%	higher
		Northern Europe	1.37	[1.28, 1.48]	100%	0%	lower
		Baltics	1.54	[1.34, 1.82]	100%	0%	average
		Mediterranean Europe	1.58	[1.47, 1.72]	100%	0%	average
	**Slightly worried**	Western Europe	1.61	[1.5, 1.73]	100%	0%	average
		Eastern Europe	1.89	[1.66, 2.15]	100%	0%	higher
		Central Europe	1.94	[1.76, 2.17]	100%	0%	higher
		South-East Europe	1.94	[1.71, 2.19]	100%	0%	higher
		Northern Europe	1.44	[1.33, 1.58]	100%	0%	lower
		Baltics	1.67	[1.41, 2.04]	100%	0%	average
		Mediterranean Europe	1.75	[1.6, 1.94]	100%	0%	average
	**Moderately worried**	Western Europe	1.82	[1.67, 2]	100%	0%	average
		Eastern Europe	2.08	[1.79, 2.45]	100%	0%	average
		Central Europe	2.16	[1.91, 2.47]	100%	0%	higher
		South-East Europe	2.21	[1.93, 2.57]	100%	0%	higher

Worried	Northern Europe	1.49	[1.37, 1.65]	100%	0%	lower
	Baltics	1.75	[1.45, 2.21]	100%	0%	average
	Mediterranean Europe	1.82	[1.65, 2.02]	100%	0%	average
	Western Europe	1.96	[1.79, 2.18]	100%	0%	average
	Eastern Europe	2.30	[1.96, 2.76]	100%	0%	average
	South-East Europe	2.48	[2.15, 2.92]	100%	0%	higher
	Central Europe	2.56	[2.24, 2.98]	100%	0%	higher
Very worried	Northern Europe	1.50	[1.38, 1.66]	100%	0%	lower
	Baltics	1.79	[1.47, 2.25]	100%	0%	average
	Mediterranean Europe	1.89	[1.71, 2.12]	100%	0%	average
	Western Europe	1.98	[1.8, 2.2]	100%	0%	average
	Eastern Europe	2.37	[2.01, 2.85]	100%	0%	average
	Central Europe	2.54	[2.21, 2.95]	100%	0%	higher
	South-East Europe	2.55	[2.2, 3]	100%	0%	higher
I don't know	Northern Europe	1.17	[1.06, 1.31]	98.7%	3.7%	average
	Central Europe	1.21	[0.92, 1.44]	88.5%	10.4%	average
	Baltics	1.23	[1.02, 1.5]	95.7%	4.7%	average
	Western Europe	1.24	[1.08, 1.4]	99%	2.3%	average
	Mediterranean Europe	1.25	[1.1, 1.41]	99.5%	1.4%	average
	Eastern Europe	1.32	[1.07, 1.64]	97.8%	2.9%	average
	South-East Europe	1.37	[1.13, 1.71]	99.3%	1.2%	average
	Northern Europe	1.47	[1.24, 1.65]	98.9%	0.6%	lower
	Baltics	1.73	[1.17, 2.25]	96.6%	0.8%	average
	Western Europe	1.79	[1.32, 2.08]	99.2%	0.5%	average
	Mediterranean Europe	1.85	[1.5, 2.13]	99.9%	0.1%	average
	Eastern Europe	2.48	[1.27, 3.15]	96.5%	0.4%	average
	Central Europe	2.54	[1.73, 3.11]	99.6%	0.1%	higher

**Missing**	South-East Europe	2.63	[1.35, 3.32]	96.9%	0.4%	higher
**Slightly severe**	Northern Europe	1.59	[1.43, 1.8]	100%	0%	lower
	Mediterranean Europe	1.69	[1.53, 1.87]	100%	0%	lower
	Baltics	1.85	[1.53, 2.31]	100%	0%	average
	Western Europe	1.91	[1.71, 2.16]	100%	0%	average
	Eastern Europe	2.40	[2.01, 2.84]	100%	0%	higher
	South-East Europe	2.41	[2.09, 2.82]	100%	0%	higher
	Central Europe	2.43	[2.11, 2.83]	100%	0%	higher
**Moderately severe**	Northern Europe	1.75	[1.54, 2.02]	100%	0%	lower
	Mediterranean Europe	1.88	[1.68, 2.13]	100%	0%	lower
	Baltics	2.12	[1.66, 2.76]	100%	0%	average
	Western Europe	2.27	[2, 2.63]	100%	0%	average
	Eastern Europe	3.06	[2.44, 3.79]	100%	0%	higher
	South-East Europe	3.08	[2.59, 3.69]	100%	0%	higher
	Central Europe	3.21	[2.73, 3.87]	100%	0%	higher
**Severe**	Northern Europe	1.82	[1.59, 2.11]	100%	0%	lower
	Mediterranean Europe	1.97	[1.74, 2.25]	100%	0%	lower
	Baltics	2.25	[1.73, 3.08]	100%	0%	average
	Western Europe	2.49	[2.16, 2.91]	100%	0%	average
	South-East Europe	3.50	[2.89, 4.28]	100%	0%	higher
	Eastern Europe	3.55	[2.8, 4.52]	100%	0%	higher
	Central Europe	3.77	[3.14, 4.65]	100%	0%	higher
	Northern Europe	1.89	[1.64, 2.22]	100%	0%	lower
	Mediterranean Europe	2.06	[1.81, 2.38]	100%	0%	lower
	Baltics	2.23	[1.63, 3.13]	100%	0%	average
	Western Europe	2.57	[2.22, 3.03]	100%	0%	average

	**Very severe**	South-East Europe	3.72	[3.05, 4.63]	100%	0%	higher
		Eastern Europe	3.88	[3.02, 5.05]	100%	0%	higher
		Central Europe	3.98	[3.27, 4.96]	100%	0%	higher
	**I don't know**	Northern Europe	1.41	[1.21, 1.63]	100%	0.2%	lower
		Mediterranean Europe	1.50	[1.33, 1.71]	100%	0%	lower
		Baltics	1.66	[1.3, 2.15]	99.7%	0.3%	average
		Western Europe	1.71	[1.49, 1.98]	100%	0%	average
		Central Europe	2.17	[1.81, 2.66]	100%	0%	higher
		South-East Europe	2.20	[1.83, 2.74]	100%	0%	higher
		Eastern Europe	2.37	[1.87, 3.19]	100%	0%	higher
	**Missing**	Northern Europe	1.71	[1.21, 2.12]	98.5%	0.8%	average
		Western Europe	1.76	[0.78, 2.48]	90.3%	2.6%	average
		Mediterranean Europe	1.86	[1.27, 2.3]	98.7%	0.7%	average
		Baltics	1.98	[1.13, 2.96]	96.7%	1%	average
		Eastern Europe	2.26	[0.34, 4.08]	84.7%	1.9%	average
		Central Europe	2.60	[1.1, 4.17]	96.2%	1.1%	average
		South-East Europe	2.61	[0.66, 4.29]	91.4%	1.2%	average
	**PrEP yes**	Mediterranean Europe	1.13	[1.1, 1.16]	100%	0%	lower
		Western Europe	1.14	[1.11, 1.17]	100%	0%	lower
		Northern Europe	1.14	[1.11, 1.18]	100%	0%	lower
		Baltics	1.16	[1.09, 1.27]	99.9%	0.5%	average
		South-East Europe	1.21	[1.06, 1.31]	98.7%	3.3%	average
		Eastern Europe	1.24	[1.12, 1.34]	99.9%	0.7%	average
		Central Europe	1.29	[1.19, 1.38]	100%	0%	higher
		South-East Europe	0.94	[0.76, 1.12]	70.9%	31.9%	average
		Baltics	0.95	[0.72, 1.05]	78.7%	43.4%	average

**PreP**							
	**Missing**	Eastern Europe	0.95	[0.77, 1.13]	68.2%	34.3%	average
		Mediterranean Europe	0.96	[0.86, 1.04]	77.6%	55.8%	average
		Western Europe	0.96	[0.85, 1.05]	75.9%	55%	average
		Central Europe	0.96	[0.77, 1.19]	62.4%	31.6%	average
		Northern Europe	0.97	[0.84, 1.05]	75%	56.1%	average
	**STI yes**	Northern Europe	1.08	[1.06, 1.11]	100%	0.9%	lower
		Mediterranean Europe	1.09	[1.07, 1.12]	100%	0.3%	lower
		Western Europe	1.10	[1.08, 1.12]	100%	0%	lower
		Baltics	1.11	[1.05, 1.19]	99.1%	4.8%	average
		Eastern Europe	1.18	[1.06, 1.26]	99%	3.3%	average
		South-East Europe	1.20	[1.12, 1.27]	100%	0.1%	higher
		Central Europe	1.22	[1.15, 1.29]	100%	0%	higher
	**STI unknown**	South-East Europe	0.95	[0.81, 1.06]	79.3%	40.4%	average
		Northern Europe	0.96	[0.84, 1.01]	90%	58%	average
		Central Europe	0.96	[0.83, 1.11]	70.2%	42.1%	average
		Mediterranean Europe	0.97	[0.9, 1.01]	87.2%	67.8%	average
		Baltics	0.97	[0.83, 1.07]	75%	56%	average
		Western Europe	0.97	[0.91, 1.03]	80.6%	75.5%	average
		Eastern Europe	0.99	[0.87, 1.15]	56.8%	44.3%	average
	**Prefer not to answer**	Western Europe	0.89	[0.69, 1.01]	93.3%	21.3%	average
		Mediterranean Europe	0.92	[0.78, 1.02]	89.5%	32.4%	average
		Central Europe	0.93	[0.65, 1.21]	67.4%	21.5%	average
		Eastern Europe	0.94	[0.58, 1.31]	61.3%	17.6%	average
		Baltics	0.94	[0.6, 1.16]	68.2%	27.6%	average
		Northern Europe	0.97	[0.79, 1.12]	62.7%	40.2%	average
		South-East Europe	1.03	[0.75, 1.36]	56.4%	19.3%	average
		Northern Europe	1.07	[0.96, 1.15]	89.4%	27.7%	average

	Mediterranean Europe	1.08	[0.92, 1.17]	84.8%	27.2%	average
	Western Europe	1.08	[0.94, 1.16]	87.3%	26.2%	average
	Baltics	1.10	[0.91, 1.25]	86.8%	18.9%	average
**Missing**	Central Europe	1.16	[0.94, 1.34]	89.9%	12.6%	average
	South-East Europe	1.18	[0.84, 1.44]	85%	10.8%	average
	Eastern Europe	1.22	[0.93, 1.5]	90.8%	8.5%	average
	Northern Europe	1.11	[1.08, 1.14]	100%	0%	lower
	Mediterranean Europe	1.14	[1.11, 1.18]	100%	0%	lower
	Western Europe	1.15	[1.12, 1.19]	100%	0%	lower
**Yes**	Baltics	1.20	[1.12, 1.33]	100%	0.1%	average
	Eastern Europe	1.33	[1.23, 1.44]	100%	0%	higher
	Central Europe	1.38	[1.3, 1.49]	100%	0%	higher
	South-East Europe	1.41	[1.32, 1.53]	100%	0%	higher
	Northern Europe	1.03	[0.99, 1.06]	89.3%	83.2%	lower
	Western Europe	1.05	[1.02, 1.09]	99.5%	47.1%	lower
	Mediterranean Europe	1.06	[1.03, 1.1]	99.9%	24.4%	average
	Baltics	1.09	[1.02, 1.18]	97%	16.5%	average
**I don't know**	South-East Europe	1.16	[1.07, 1.25]	99.8%	2.7%	average
	Central Europe	1.19	[1.11, 1.28]	100%	0.1%	higher
	Eastern Europe	1.19	[1.1, 1.3]	100%	0.2%	higher
	South-East Europe	0.80	[0.59, 1.08]	89.2%	10.6%	average
	Western Europe	0.80	[0.63, 0.93]	99.8%	2.1%	average
	Eastern Europe	0.81	[0.61, 1.09]	87.6%	11.8%	average
	Baltics	0.85	[0.6, 1.07]	88.8%	12.9%	average
**Prefer not to answer**	Central Europe	0.86	[0.66, 1.12]	82.2%	15.7%	average
	Mediterranean Europe	0.87	[0.75, 0.96]	98.9%	7.5%	average
	Northern Europe	0.90	[0.76, 1.02]	91.6%	20.4%	average

	Central Europe	0.29	[0.072, 0.88]	97%	1.3%	lower
	Eastern Europe	0.93	[0.21, 1.55]	56%	8.6%	average
	Baltics	0.94	[0.29, 1.31]	57.2%	12.7%	average
**Missing**	Mediterranean Europe	1.06	[0.9, 1.18]	75.5%	34.9%	average
	Northern Europe	1.14	[0.98, 1.21]	93.1%	9.5%	average
	Western Europe	1.20	[1.11, 1.27]	99.8%	0.8%	average
	South-East Europe	1.22	[0.32, 1.83]	64.3%	5.9%	average

S9. Association plot

In this figure are shown the posterior RR distributions of the associations between the factors of interest and the MPX vaccine acceptance, divided by type of global estimate (country/subregion adjusted and unadjusted). Also, the estimates at the level of each European subregion are presented. The bars for each factor value represent the 90% CrI (with horizontal markers at 50% and 90% levels). The global estimates are distinguished by a triangle while the subregional ones by points.
For readibility reasons, the plot for the impact of the region of origin/birth of migrants is plotted separetely.

General plot

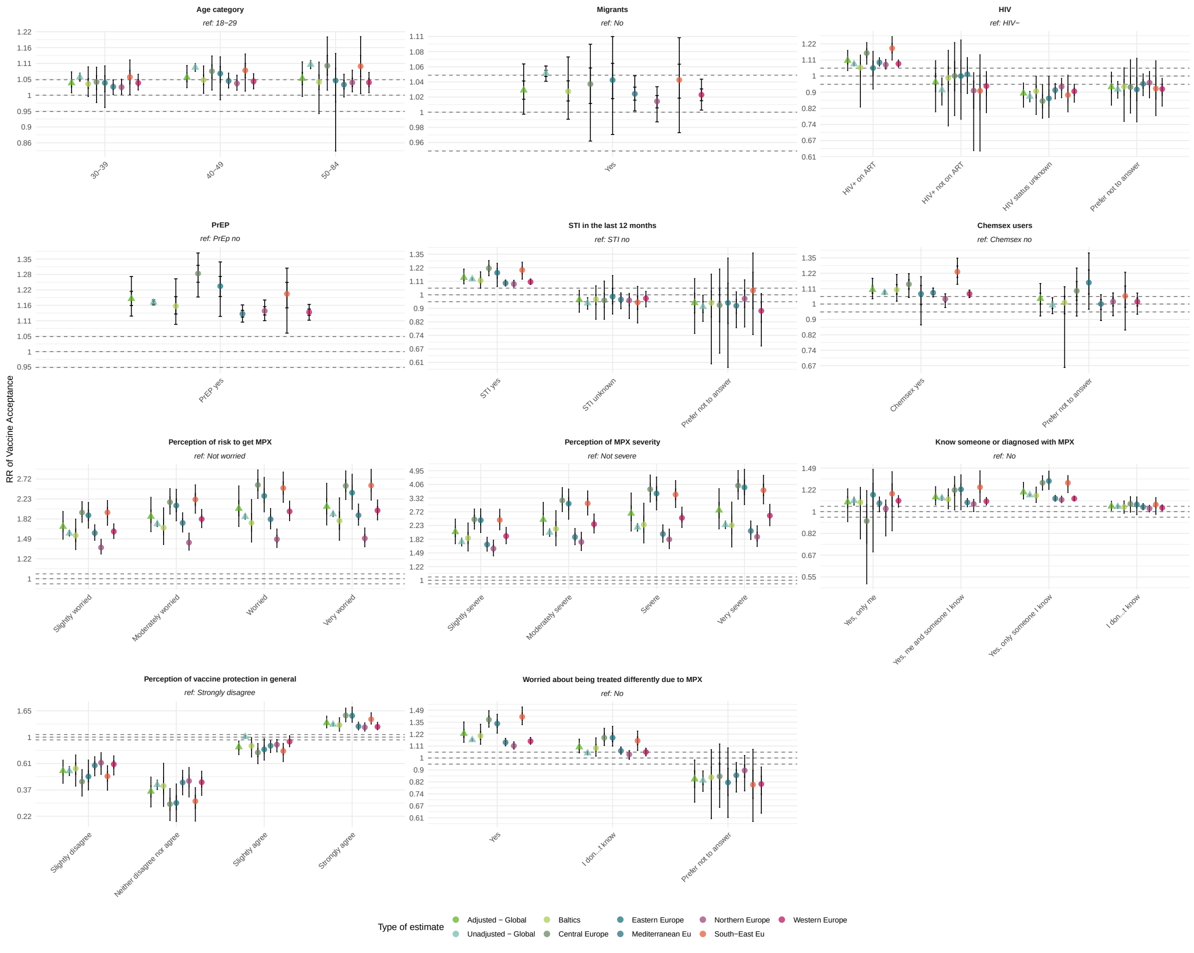

Migrant birth region plot

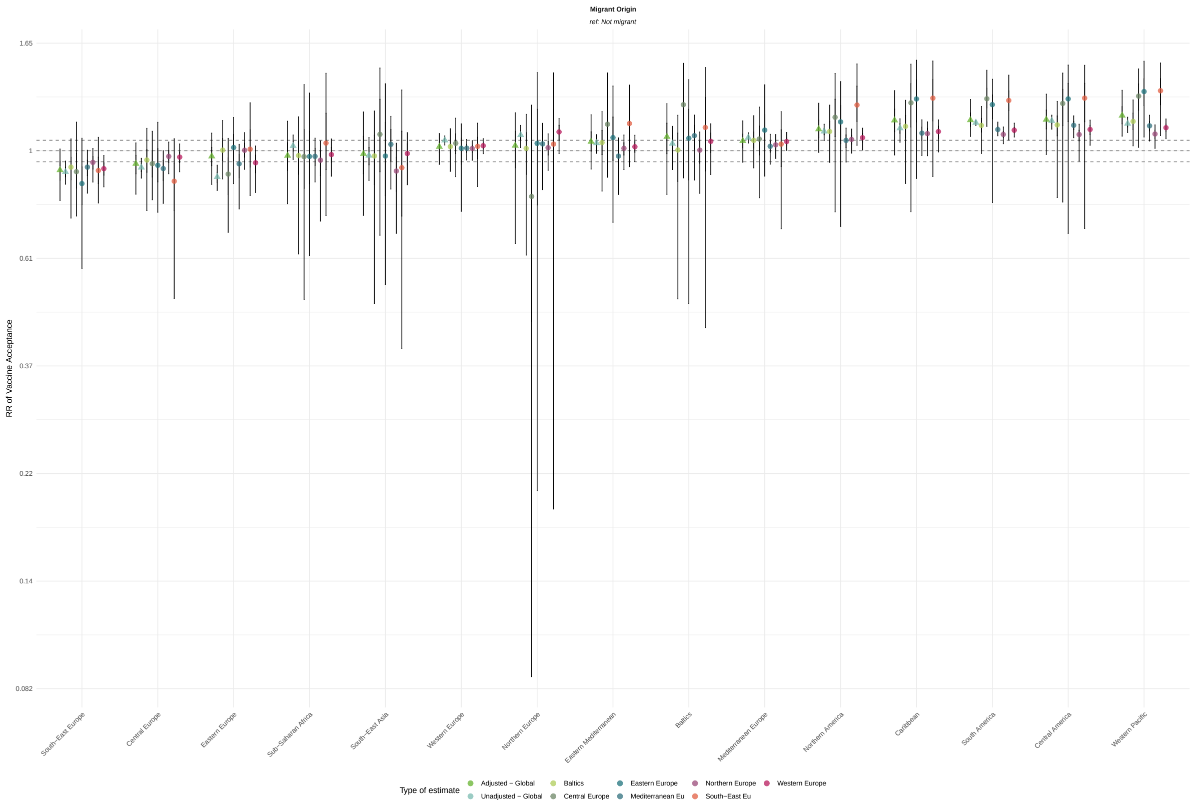

S10. Session Info

R version 4.2.0 (2022-04-22)

Platform: aarch64-apple-darwin20 (64-bit)

locale: en_US.UTF-8||en_US.UTF-8||en_US.UTF-8||C||en_US.UTF-8||en_US.UTF-8

attached base packages: *parallel*, *stats*, *graphics*, *grDevices*, *utils*, *datasets*, *methods* and *base*

other attached packages: *pander(v.0.6.5)*, *knitr(v.1.40)*, *kableExtra(v.1.3.4)*, *brms(v.2.17.0)*, *Rcpp(v.1.0.8.3)*, *here(v.1.0.1)*, *rlang(v.1.0.4)*, *readxl(v.1.4.0)*, *purrr(v.0.3.4)*, *tidyr(v.1.2.0)*, *stringr(v.1.4.0)*, *readr(v.2.1.2)*, *ggplot2(v.3.3.6)* and *dplyr(v.1.0.9)*

loaded via a namespace (and not attached): *backports(v.1.4.1)*, *Hmisc(v.4.7-0)*, *systemfonts(v.1.0.4)*, *plyr(v.1.8.7)*, *igraph(v.1.3.1)*, *splines(v.4.2.0)*, *crosstalk(v.1.2.0)*, *rstantools(v.2.2.0)*, *inline(v.0.3.19)*, *digest(v.0.6.29)*, *htmltools(v.0.5.2)*, *fansi(v.1.0.3)*, *magrittr(v.2.0.3)*, *checkmate(v.2.1.0)*, *cluster(v.2.1.3)*, *tzdb(v.0.3.0)*, *RcppParallel(v.5.1.5)*, *matrixStats(v.0.62.0)*, *vroom(v.1.5.7)*, *xts(v.0.12.1)*, *svglite(v.2.1.0)*, *prettyunits(v.1.1.1)*, *jpeg(v.0.1-9)*, *colorspace(v.2.0-3)*, *rvest(v.1.0.2)*, *xfun(v.0.31)*, *callr(v.3.7.0)*, *crayon(v.1.5.1)*, *jsonlite(v.1.8.0)*, *survival(v.3.3-1)*, *zoo(v.1.8-10)*, *glue(v.1.6.2)*, *gtable(v.0.3.0)*, *emmeans(v.1.7.5)*, *webshot(v.0.5.3)*, *distributional(v.0.3.0)*, *pkgbuild(v.1.3.1)*, *rstan(v.2.21.5)*, *abind(v.1.4-5)*, *scales(v.1.2.0)*, *mvtnorm(v.1.1-3)*, *DBI(v.1.1.3)*, *miniUI(v.0.1.1.1)*, *viridisLite(v.0.4.0)*, *xtable(v.1.8-4)*, *htmlTable(v.2.4.0)*, *bit(v.4.0.4)*, *foreign(v.0.8-82)*, *Formula(v.1.2-4)*, *stats4(v.4.2.0)*, *StanHeaders(v.2.21.0-7)*, *DT(v.0.23)*, *htmlwidgets(v.1.5.4)*, *httr(v.1.4.3)*, *threejs(v.0.3.3)*, *RColorBrewer(v.1.1-3)*, *posterior(v.1.2.1)*, *ellipsis(v.0.3.2)*, *pkgconfig(v.2.0.3)*, *loo(v.2.5.1)*, *farver(v.2.1.0)*, *nnet(v.7.3-17)*, *utf8(v.1.2.2)*, *tidyselect(v.1.1.2)*, *reshape2(v.1.4.4)*, *later(v.1.3.0)*, *munsell(v.0.5.0)*, *cellranger(v.1.1.0)*, *tools(v.4.2.0)*, *cli(v.3.3.0)*, *generics(v.0.1.3)*, *EcdcColors(v.0.0.0.9000)*, *ggridges(v.0.5.3)*, *evaluate(v.0.15)*, *fastmap(v.1.1.0)*, *yaml(v.2.3.5)*, *bit64(v.4.0.5)*, *processx(v.3.5.3)*, *nlme(v.3.1-157)*, *mime(v.0.12)*, *xml2(v.1.3.3)*, *compiler(v.4.2.0)*, *bayesplot(v.1.9.0)*, *shinythemes(v.1.2.0)*, *rstudioapi(v.0.13)*, *png(v.0.1-7)*, *tibble(v.3.1.8)*, *stringi(v.1.7.8)*, *ps(v.1.7.0)*, *Brobdingnag(v.1.2-7)*, *forcats(v.0.5.1)*, *lattice(v.0.20-45)*, *Matrix(v.1.4-1)*, *markdown(v.1.1)*, *shinyjs(v.2.1.0)*, *tensorA(v.0.36.2)*, *vctrs(v.0.4.1)*, *pillar(v.1.8.1)*, *lifecycle(v.1.0.1)*, *bridgesampling(v.1.1-2)*, *estimability(v.1.3)*, *data.table(v.1.14.2)*, *httpuv(v.1.6.5)*, *R6(v.2.5.1)*, *latticeExtra(v.0.6-29)*, *promises(v.1.2.0.1)*, *gridExtra(v.2.3)*, *codetools(v.0.2-18)*, *colourpicker(v.1.1.1)*, *gtools(v.3.9.2.1)*, *assertthat(v.0.2.1)*, *rprojroot(v.2.0.3)*, *withr(v.2.5.0)*, *shinystan(v.2.6.0)*, *hms(v.1.1.1)*, *grid(v.4.2.0)*, *rpart(v.4.1.16)*, *coda(v.0.19-4)*, *rmarkdown(v.2.14)*, *shiny(v.1.7.1)*, *base64enc(v.0.1-3)* and *dygraphs(v.1.1.1.6)*

References

Bürkner, Paul-Christian. 2017. "Brms: An r Package for Bayesian Multilevel Models Using Stan." *Journal of Statistical Software* 80: 1–28.

Carpenter, Bob, Andrew Gelman, Matthew D Hoffman, Daniel Lee, Ben Goodrich, Michael Betancourt, Marcus Brubaker, Jiqiang Guo, Peter Li, and Allen Riddell. 2017. "Stan: A Probabilistic Programming Language." *Journal of Statistical Software* 76 (1).

Makowski, Dominique, Mattan S Ben-Shachar, and Daniel Lüdecke. 2019. "bayestestR: Describing Effects and Their Uncertainty, Existence and Significance Within the Bayesian Framework." *Journal of Open Source Software* 4 (40): 1541.

R Core Team. 2022. *R: A Language and Environment for Statistical Computing.* Vienna, Austria: R Foundation for Statistical Computing. https://www.R-project.org/.
